# Prenylated Flavonoids and C-15 Isoprenoid Analogues with Antibacterial Properties from the Whole Plant of *Imperata cylindrica* (L.) Raeusch (Gramineae)

**DOI:** 10.3390/molecules26164717

**Published:** 2021-08-04

**Authors:** Romeo D. Tadjouate Nago, Paul Nayim, Armelle T. Mbaveng, James D. Simo Mpetga, Gabin T. Mbahbou Bitchagno, Badawe Garandi, Pierre Tane, Bruno N. Lenta, Norbert Sewald, Mathieu Tene, Victor Kuete, Augustin Silvere Ngouela

**Affiliations:** 1 Department of Chemistry, Faculty of Science, University of Dschang, P.O. Box 67 Dschang, Cameroon; nromeodesiae@yahoo.fr (R.D.T.N.); mtene2001@yahoo.fr (M.T.); sngouela@yahoo.fr (A.S.N.); 2 Department of Biochemistry, Faculty of Science, University of Dschang, P.O. Box 67 Dschang, Cameroon; nayimpaul@yahoo.fr (P.N.); armkuete@yahoo.fr (A.T.M.); Badawegarandi2@gmail.com (B.G.); kuetevictor@yahoo.fr (V.K.); 3 Organic and Bioorganic Chemistry, Department of Chemistry, Bielefeld University, D-33501 Bielefeld, Germany; norbert.sewald@uni-bielefeld.de; 4 Department of Chemistry, Higher Teacher Training College, University of Yaounde I, P.O. Box 47 Yaounde, Cameroon; lentabruno@yahoo.fr

**Keywords:** *Imperata cylindrica*, Gramineae, flavonoids, terpenoids, antibacterial properties

## Abstract

The local botanical *Imperata cylindrica* in Cameroon was investigated for its antibacterial potency. The methanol extract afforded a total of seven compounds, including five hitherto unreported compounds comprising three flavonoids (**1**–**3**) and two C-15 isoprenoid analogues (**4** and **5**) together with known derivatives (**6** and **7**). The novelty of the flavonoids was related to the presence of both methyl and prenyl groups. The potential origin of the methyl in the flavonoids is discussed, as well as the chemophenetic significance of our findings. Isolation was performed over repeated silica gel and Sephadex LH-20 column chromatography and the structures were elucidated by (NMR and MS). The crude methanol extract and isolated compounds showed considerable antibacterial potency against a panel of multi-drug resistant (MDR) bacterial strains. The best MIC values were obtained with compound (**2**) against *S. aureus* ATCC 25923 (32 µg/mL) and MRSA1 (16 µg/mL).

## 1. Introduction

Infectious diseases cause 15 million deaths each year, accounting for approximately 27.12% of deaths worldwide [1]. The rapid emergence of multi-drug resistant bacteria has severely compromised the efficacy of antibiotics and, hence, the fight against bacterial infections worldwide [2]. The search for new compounds to tackle bacterial resistance has become imperative. African flora is a rich source of bioactive substances, and several medicinal plants have proven their ability to inhibit the growth of Gram-negative bacteria [3,4,5,6]. The present study focusses on *Imperata cylindrica* (Gramineae), an aggressive grass endemic to tropical and subtropical Asia, Australia, Africa, and southern Europe [7,8,9]. This plant species has different names in various languages around the World, such as “speargrass” in Nigeria, “alang-alang” in Asia, “cogongrass” in America, or “Ngnig-gah” in Western Cameroon, amongst others [7]. *I. cylindrica* can grow up to 1.2 m high with narrow and rigid leaves, bearded at the base and glabrous in their upper part [10]. It adversely affects agroforestry operations because it suppresses the growth of trees and crop plants and is a fire hazard [7,11,12,13]. *I. cylindrica* constitutes one of the most reputed species of the genus after some species previously thought to be members of this genus were reassigned to other groups of plants. It is reputed in traditional medicine for the treatment of hemorrhage, improvement of urination, and immunostimulation [14]. The rhizomes of this plant are advised by traditional healers as a diuretic and are used to treat inflammations and fever in Korean herbal medicine [15]. Investigations carried out on this plant species have revealed antibacterial and anticancer activities [16,17]. Furthermore, the root extract of *I. cylindrica* is non-toxic upon short and long-term oral administration [18]. The chemistry and pharmacology of *I. cylindrica* are diverse and rich. The plant contains coumarins and chromones [19], lignans [20], methoxylated flavonoids [19], phenolic acids [21], and steroids [22]. Crude extracts and isolated compounds from this plant showed a broad band of biological potency including cytotoxic, neuroprotective, and vasodilatory effects [20]. Within the frame of our ongoing research into the characterization of bioactive metabolites from Cameroonian folk medicine [23,24,25,26,27,28], we report herein the occurrence of five unprecedented compounds in nature with antibacterial properties.

## 2. Results

**Figure 1:**
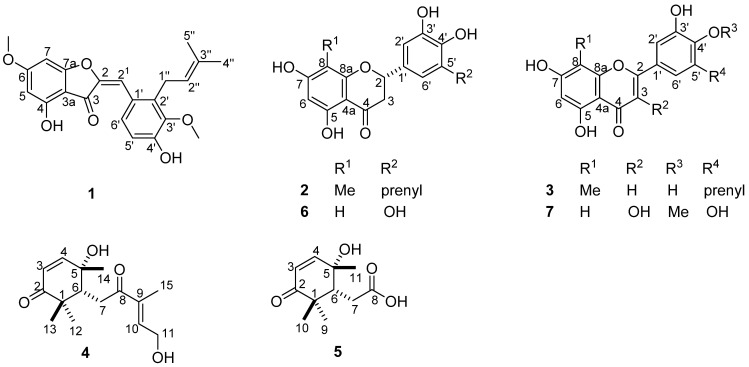
Chemical structures of isolated compounds (**1**–**7**) from *I. cylindrica*.

Successive column chromatography of the MeOH extract obtained from the whole plant *Imperata cylindrica* over silica gel and Sephadex LH-20 afforded three previously undescribed flavonoids (**1**–**3**) and two new C-15 isoprenoid analog (**4** and **5**) together with readily available flavonoids (**6** and **7**) and a mixture of phytosterols (Figure 1). Their structures were determined by spectroscopic and spectrometric data.

Compound **1** was isolated as an orange powder and its molecular formula C_22_H_22_O_6_ was deduced from HRESI-MS, which showed the pseudomolecular ion [M + Na]^+^ at *m*/*z* 405.1300 (calcd. *m*/*z* 405.1308 for C_22_H_22_O_6_Na^+^). The IR spectrum showed absorption bands at 3306 (O–H), 2923 (C–H), 1709 (C=O), 1610 (C=C), and 1069 (C–O) cm^−1^. The NMR spectra (Table 1) of **1** exhibited two meta-coupled aromatic resonances at *δ_H_*/*δ*_C_ 6.38 (1H, d, *J* = 2.1 Hz, H-7)/90.2 (C-7) and 6.13 (1H, d, *J* = 2.1 Hz, H-5)/97.7 (C-5); a one-proton singlet at *δ_H_* 6.90 (1H, s, H-2^1^) attributable to aurone-type flavonoids [29,30]; and aromatic AB-type resonances at *δ_H_*/*δ*_C_ 7.93 (1H, d, *J* = 8.2 Hz, H-6′)/129.5 (C-6′) and 6.83 (1H, d, *J* = 8.2 Hz, H-5′)/115.5 (C-5′).

**Figure 2:**
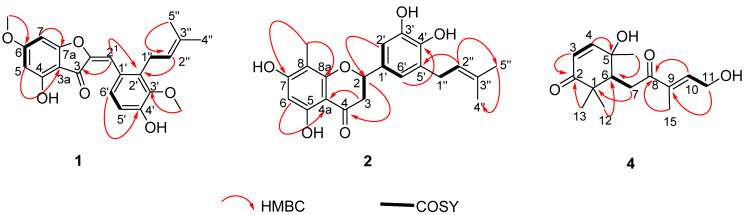
Diagnostic HMBC and COSY correlations of **1**, **2**, and **4**.

The meta-coupled protons belonged to ring A of the aurone core skeleton, and HMBC interactions (Figure 2) were observed from individual protons H-5 to carbons at *δ*_C_ 170.4 (C-4), 105.5 (C-3a), and 90.2 (C-7) and from H-7 to carbons at *δ*_C_, 159.3 (C-7a), 169.2 (C-6), and 97.7 (C-5). Other HMBC interactions were also evidenced from H-6′ to carbons at *δ*_C_ 124.0 (C-1′), 138.3 (C-2′), 147.0 (C-4′), and to the aurone olefinic carbon at 109.7 (C-2^1^) amongst others. While H-2^1^ also showed cross peaks for C-6′, C-1′, and C-2′, an aliphatic methylene at *δ_H_*/*δ*_C_ 3.55 (H-1′’)/26.3 (C-1′’) and a second olefinic proton at *δ_H_*/*δ*_C_ 5.09 (H-2′’)/124.7 (C-2′’), part of a prenyl substituent borne by carbon C-2′, evidenced HMBC interactions with carbons C-1′ (*δ*_C_ 124.0), C-2′ (*δ*_C_ 138.3), and C-3′ (*δ*_C_ 148.1). Further resonances of the prenyl moiety were visible at *δ*_C_ 132.9 for the quaternary C-3″ and at *δ_H_*/*δ*_C_ 1.70 (3H, s, H-4″)/25.9 (C-4″) and 1.90 (3H, s, H-5″)/18.2 (C-5″) for the geminal dimethyl. Two methoxyl groups resonate at *δ_H_*/*δ*_C_ 3.78/61.2 and 3.90/56.5, and were positioned at C-3ʹ and C-6 based on their proton to neighboring carbons long-range correlations with C-3′ (*δ*_C_ 148.1) and C-6 (*δ*_C_ 169.2), respectively. Prenylated aurones were previously reported in the literature without the methoxyl groups as encountered in **1** [31]. Compound **1** was then characterized as a new aurone derivative for which we proposed the trivial name cylindraucine.

Compound **2** was obtained as a yellow powder and its molecular formula C_21_H_22_O_6_ was deduced from the HRESI-MS, which showed the pseudo-molecular ion [M + H]^+^ at *m*/*z* 371.1478 (calcd. *m*/*z* 371.1489 for C_21_H_23_O_6_^+^). The IR spectrum showed absorption bands at 3233 (O–H), 2921 (C–H), 1634 (C=O), 1600 (C=C), and 1104 (C–O) cm^−1^. Its NMR spectra (Table 2) exhibited AX-type aromatic resonances at *δ_H_*/*δ*_C_ 6.78 (1H, d, *J* = 2.2 Hz, H-2′)/111.7 (C-2′) and 6.69 (1H, d, *J* = 2.2 Hz, H-6′)/119.7 (C-6′), sharing the same benzene ring with a prenyl moiety as proven by HMBC cross peaks from the methylene H-1′’ (*δ_H_* 3.32) to C-6′ (*δ*_C_ 119.7), C-5′ (*δ*_C_ 129.5), C-4′ (*δ*_C_ 144.4), and C-2′’ (*δ*_C_ 123.7). The NMR spectra also evidenced another aromatic one-proton singlet at *δ_H_*/*δ*_C_ 5.95 (1H, s, H-6)/95.2 (C-6), adjacent to two phenol-type groups, and a methyl group at *δ_H_*/*δ*_C_ 1.96 (3H, s, H-1′′’)/6.9 (C-1′’′). The later protons showed HMBC contacts to carbons C-8 (*δ*_C_ 105.2), C-8a (*δ*_C_ 162.1), and C-7 (*δ*_C_ 166.0), while the former showed interactions to carbons C-4 (*δ*_C_ 197.7), C-7 (*δ*_C_ 166.0), C-5 (*δ*_C_ 162.4), C-8 (*δ*_C_ 105.2), and C-4a (*δ*_C_ 103.0). Both aromatic moieties were connected through a three-carbon chain comprising the carbonyl C-4, the aliphatic methylene at *δ_H_* 3.04 (H-3a)/2.70 (H-3b)/*δ*_C_ 44.1 (C-3), and an oxymethine at *δ_H_* 5.23 (H-2)/*δ*_C_ 80.5 (C-2). Compound **2** was then characterized as a flavanone-type flavonoid bearing a prenyl at C-5′ and an upfield methyl at C-8. H-2 and H-3a showed a *trans*-arrangement as judged by their mutual coupling constant *J* = 12.5 Hz [24,32,33] characteristic of a *trans*-diaxial relationship between H-2 and H-3a. The phenyl group at C-2 was α-oriented following the comparison of the chemical shifts of carbons C-2, C-3, and C-1′ with those of the literature [32,33].

Likewise, compound **3** was isolated in the form of a yellow powder and its molecular formula C_21_H_20_O_6_ was deduced from its HRESI-MS, which showed the sodium adduct [M + Na]^+^ at *m*/*z* 391.1140 (calcd. *m*/*z* 391.1152 for C_21_H_20_O_6_Na^+^). The IR spectrum showed absorption bands at 3355 (O–H), 2922 (C–H), 1642 (C=O), 1600 (C=C), and 1087 (C–O) cm^−1^. The NMR spectra of **3** (Table 2) were similar to those of compound **2** except the lack of resonances attributable to characteristic aliphatic signals of flavanones and rather the appearance of a one-proton singlet at *δ*_H_/*δ*_C_ 6.55 (1H, s, H-3)/113.3 (C-3) in compound **3**. Moreover, each of the meta-coupled protons H-2′ and H-6′, as well as the olefinic proton H-3, showed HMBC cross peaks to an oxygenated olefinic carbon at *δ*_C_ 146.1 (C-2). These resonances identified compound **3** as a flavone [24,34].

Compounds **2** and **3** are unique in nature because of the upfield methyl group encountered at C-8 in both structures paired with the prenyl substituent at C-5′. Gancaonin E, a known compound related both to **2** and **3**, is prenylated at the C-8 position [35]. The methyl group at C-8 in our derivatives might originate from the oxidation of the prenyl substituent in related analogues. Various flavonoids containing methyl groups in either position C-6 or C-8 have been isolated [33]. Thus, **2** and **3** are newly characterized and we proposed the trivial names cylindricines A (**2**) and B (**3**), respectively.

Compound **4** was obtained as a brown oil and its molecular formula C_15_H_22_O_4_ was deduced from its HRESI-MS, which exhibited the sodium adduct [M + Na]^+^ at *m*/*z* 289.1407 (calcd. *m*/*z* 289.1410 for C_15_H_22_O_4_Na^+^). The IR spectrum showed absorption bands at 3377 (O-H), 2977 (C-H), 1707 (C = O), 1663 (C = C), and 1060 (C-O) cm^−1^. Its NMR spectra (Table 1) showed signals corresponding to geminal dimethyl groups at *δ*_H_/*δ*_C_ 1.01 (s, H-12)/25.0 (C-12) and 1.07 (s, H-13)/21.8 (C-13), proved by HMBC contacts to carbons C-2 (*δ*_C_ 205.8), C-1 (*δ*_C_ 46.8), C-6 (*δ*_C_ 49.6), and to one another. The carbonyl C-2 is conjugated to an olefin at *δ*_H_/*δ*_C_ 6.78 (1H, d, *J* = 10.3 Hz, H-4)/156.2 (C-4) and 5.82 (1H, d, *J* = 10.3 Hz, H-3)/124.1 (C-3) as judged by HMBC cross peaks from H-4 to C-2 or from H-3 to C-1. The olefin was also adjacent to a hydroxylated quaternary carbon at *δ*_C_ 71.9 (C-5), bearing a methyl group as evidenced by HMBC contacts from the methyl to nearby resonances C-4 (*δ*_C_ 156.2), C-5 (*δ*_C_ 71.9), and C-6 (*δ*_C_ 49.6). The COSY spectrum evidenced square correlations between the methine H-6 (*δ*_H_ 2.86, m) and the methylene protons H-7a (*δ*_H_ 2.99, dd, *J* = 15.9, 6.1 Hz) and H-7b (*δ*_H_ 2.88, dd, *J* = 15.9, 6.1 Hz). Both groups of protons also showed HMBC contacts to a second α, β-unsaturated carbonyl moiety at *δ*_C_ 203.4 (C-8), 140.9 (C-10), and 137.5 (C-9), bearing a methyl at *δ*_H_/*δ*_C_ 1.75 (s, H-15)/10.9 (C-15) and a hydroxymethylene at *δ*_H_/*δ*_C_ 4.36 (d, *J* = 5.7 Hz, H-11)/58.9 (C-11) judged by HMBC contacts between carbons C-9, C-8, and C-10.

**Figure 3:**
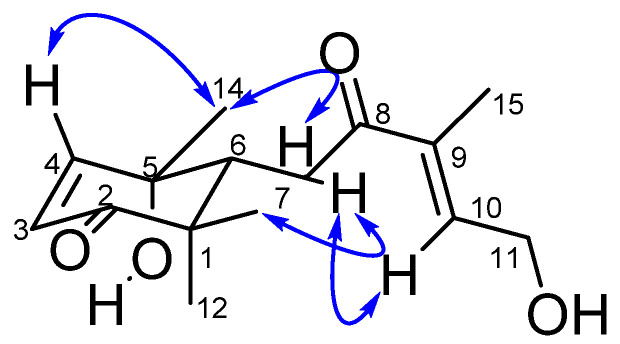
Some ROESY correlations of **4**.

The relative stereochemistry of the stereocenters and olefins was established based on the coupling constants and ROESY cross peaks (Figure 3) of **4**. H-4 and H-3 showed a *cis* arrangement with *J* = 10.3 Hz as further suggested by the absence of ROESY interaction between these protons. In addition, the ROESY spectrum showed interactions from the methyl H-14 to H-7a but to none of the geminal dimethyl at C-1, indicative of the equatorial position of the methyl C-14. The β-position of H-6 was confirmed by cross peaks visible from H-7b to the β-methyl H-13 and from H-6 to H-13 and H-7b. The interaction from H-7b to the olefin H-10 indicated a δ-transoid conformation of the α, β-unsaturated ketone at C-8 with a *cis*-relationship of the methyl and hydroxymethylene groups as illustrated by ROESY cross peaks from H-15 to H-11. Compound **4** was thus characterized as 3*Z*, 9*E*, 5*S*, 6*R*, similar to reported analogues in the literature [36,37]. Compound **4** was thus a new C-15 isoprenoid analogue related to abscisic acid, a well-known plant hormone involved in abscission [37]. We proposed the trivial name cylindracid A (**4**).

Compound **5** was obtained as a brown oil and its molecular formula C_11_H_16_O_4_ was deduced from its HRESI-MS, which exhibited the pseudo-molecular ion [M−H]^−^ at *m*/*z* 211.0985 (calcd. *m*/*z* 211.0975 for C_11_H_15_O_4_^−^). The NMR spectra profiles of **5** (Table 1) were similar to those of compound **4** except the lack of resonances of the prenyl group attached to C-7 and the appearance of a carboxylic group resonance at *δ*_C_ 177.5 (C-8) in compound **5**. Compound **5** could be derived from its parent compound **4** by oxidation of the side prenyl chain at C-7. Its stereochemistry was found to be similar to that of compound **4**, the angular methyl C-11, and the new carboxylic group, with both being equatorially oriented. Compound **5** was thus newly characterized and we proposed the trivial name cylindracid B. The corresponding IR, MS and NMR spectra of each of the compounds described here are available as supplementary material (Figures S1–S38).

While compounds **4** and **5** were isolated in low amounts and thus some signals were missing in their respective ^1^H-NMR and ^13^C-NMR spectra, the signal assignment for both compounds could be completed by using the respective spectra of the mixture of **4** and **5**. The known compounds were identified as 3’,4’,5,5’,7-pentahydroxyflavanone (**6**) [38] and mearnsetin (**7**) [39] by comparison of their NMR and MS data with those reported in the literature. A mixture of stigmasterol and β-sitosterol was also isolated and identified by comparison of the analytical TLC and IR spectra with a sample from our laboratory.

As shown by the results presented in Table 3, the crude methanol extract of *I. cylindrica,* as well as the isolated compounds, inhibited the growth of all the tested Gram-positive and Gram-negative microorganisms. Depending on the microorganism, the MIC values of the crude extract ranged from 256 to 1024 μg/mL, while the MIC values of the pure compounds ranged from 128 to 1024 μg/mL (**1**), 16 to 128 μg/mL (**2**), 64 to 256 μg/mL (**3**), 512 to 1024 μg/mL (**4**), 64 to 512 μg/mL (**5**), and 32 to 128 μg/mL (**6**).

## 3. Discussion

This study reports on the characterization of seven compounds, including five flavonoids (**1**–**3**, **6** and **7**) and two C-15 isoprenoid analogues (**4** and **5**). Compounds **1**–**3** are prenylated flavonoids and are identified and described for the first time in this study. This is the first report of prenylated flavonoids from the genus *Imperata*. Compounds **4** and **5** are also new and have been identified for the first time. Compounds **6** and **7** were readily known but are isolated and described for the first time from the family Gramineae. Nonetheless, non-prenylated flavonoids have already been reported from *Imperata* species [19,22,40]. *Imperata cylindrica* has a very high molecular diversity, scanning methoxylated flavonoids [19], coumarins and chromones [19], lignans [8,20], phenolic acids [21], and terpenoids [41,42]. The most encountered groups of flavonoids are non-prenylated and unmethylated at position 8. The prenylated flavonoids described in this work are isolated for the first time in the genus *Imperata*. The terpenoids most often found in *Imperata cylindrica* or in the genus *Imperata* are triterpenes. However, a sesquiterpene has already been isolated from *Imperata* [20]. Compounds **4** and **5** isolated and described herein enrich the chemotaxomia of the plant. Our results confirm the molecular diversity observed in the literature for the genus *Imperata*.

Following the definition by [4], the crude methanol extract of *I. cylindrica* showed moderate antibacterial activity against almost all the tested bacteria strains. Compounds **1** and **4** displayed weak activities toward all the tested microorganisms while compound **3** moderately inhibited the growth of *E. aerogenes* ATCC 13048 and *S. aureus* MRSA1. Compound **5** exerted moderate activity on *E. aerogenes* ATCC 13048, comparable to the activity obtained with compound **6** against *E. coli* AG102 and *S. aureus* ATCC 25923. The best activity of compound **2** was obtained against *S. aureus* ATCC 25923 and MRSA1. The known antimicrobial mechanisms associated with plant secondary metabolites [43] may explain the antibacterial effect of isolated compounds from *I. cylindrica.* Although the antimicrobial activity of the methanol extract of *I. cylindrica* was previously demonstrated against a panel of Gram-positive MDR bacteria [16], to the best of our knowledge, the chemical and antibacterial profiling of compounds from *I. cylindrica* against MDR microorganisms is being reported here for the first time. This study supports the antimicrobial traditional use of *I. cylindrica.*

## 4. Materials and Methods

### 4.1. General Experimental Procedures

Electrospray ionization (ESI) mass spectra were recorded on a 1200-series HPLC-system or a 1260-series Infinity II HPLC-system (Agilent-Technologies, Santa Clara, CA, USA) with binary pump and integrated diode array detector coupled to an LC/MSD-Trap-XTC-mass spectrometer (Agilent-Technologies) or an LC/MSD Infinity lab LC/MSD (G6125B LC/MSD). High resolution mass spectra were recorded on a Micromass-Q-TOF-Ultima-3-mass spectrometer (Waters, Milford, MA, USA) with LockSpray-interface and a suitable external calibrant. Infrared (IR) spectra were recorded on a FTIR-spectrometer (Bruker Tensor 27, Billerica, MA, USA) equipped with a diamond ATR unit and are reported in terms of frequency of absorption in cm^−1^. NMR spectra were recorded on a Bruker Avance III 500 HD (^1^H: 500 MHz, ^13^C: 125 MHz) or Avance 600 (^1^H-NMR: 600 MHz and ^13^C-NMR: 151.1 MHz). Chemical shifts *δ* (ppm) are reported relative to residual solvent signal. 2D spectra (COSY, HMQC, HMBC) and DEPT-135 spectra were used for signal assignment. Chromatographic purification of compounds was performed on silica gel (35–70 μm, Acros Organics, Fair Lawn, NJ, USA) and Sephadex LH-20. Thin-layer chromatography (TLC) was carried out on silica plates (TLC Silica 60 F_254_ by Merck, Kenilworth, NJ, USA) and spots were detected by spraying with 20% H_2_SO_4_ followed by charring at 100 °C.

### 4.2. Plant Material

The whole plant of *Imperata cylindrica* was collected at Dschang in the western Region of Cameroon in April 2020. The identification was performed at the Cameroon National Herbarium (Yaoundé, Cameroon) by comparison with the voucher specimen kept under the voucher number 30139/SRF-Cam.

### 4.3. Extraction and Purification

Dried materials of *Imperata cylindrica* (1.5 kg) were ground and extracted with methanol (3 × 5 L, 72 h each) at room temperature to yield a semi-solid crude extract (120.0 g) after removal of the solvent under reduced pressure. A portion (104.0 g) of this extract was flashed over an opened silica gel column chromatography with gradients of *n*-hexane-EtOAc then EtOAc-MeOH. Seventy-four fractions of 200 mL each were collected and combined on the basis of their TLC profiles (using mixtures of *n*-hexane-EtOAc 85:15, 70:30, 30:70) into six main fractions coded FA–FF (FA: 1–13; FB: 14–21; FC: 22–32; FD: 33–45; FE: 46–55, FF: 56–74). Fraction FA (26.3 g) contained mostly lipids and was not further investigated. Fraction FB (9.4 g) was submitted to silica gel column chromatography eluted with gradients of *n*-hexane-EtOAc. Fractions of 75 mL each were collected, evaporated under reduced pressure, and combined on the basis of their analytical TLC profiles, affording three sub-fractions FB1–FB3. Sub-fraction FB3 (1.3 g) was further purified on a Sephadex LH-20 column chromatography with isocratic MeOH yielding compound **1** (Hex/EtOAc 80:20, retention factor *k* 2.06, 22.6 mg). Fraction FC (17.1 g) was eluted over a silica gel column chromatography with gradients of *n*-hexane-EtOAc. Fractions of 75 mL each were collected, dried, and combined on the basis of their analytical TLC to afford five sub-fractions FC1–FC5. Sub-fractions FC1 (2.0 g), FC2 (1.4 g), and FC4 (3.1 g) were purified each over repeated Sephadex LH-20 column chromatography eluted with MeOH to give compounds **2** (Hex/EtOAc 70:30, *k* 3.44, 10.1 mg) and **3** (Hex/EtOAc 70:30, *k* 4.00, 13.2 mg), respectively. Fractions FD (6.7 g) and FE (5.2 g) precipitated partially in EtOAc, yielding a yellow powder, compounds **6** (Hex/EtOAc 60:40, *k* 5.00, 16.7 mg) and **7** (Hex/EtOAc 60:40, *k* 5.90, 9.8 mg), respectively, upon filtration. Further investigations of these fractions were not conclusive. Fraction FF (30.2 g) was also purified as previously described over a silica gel column chromatography with gradients of *n*-hexane-EtOAc. Fractions of 100 mL each were collected, dried under reduced pressure, and combined using analytical TLC, leading to three sub-fractions FF1–FF3. Sub-fraction FF2 (2.8 g) was purified on repeated Sephadex LH-20 column chromatography eluted with MeOH to give a mixture of compounds **4** and **5** (10.2 mg, oil). The purification of the latter by silica gel column chromatography with gradients of CH_2_Cl_2_/MeOH afforded **4** (Hex/EtOAc 20:80, *k* 8.40, 4.0 mg) and **5** (Hex/EtOAc 20:80, *k* 8.7, 1.5 mg).

### 4.4. Cylindraucine (***1***)

Orange amorphous powder; IR (ν_max_) 3306, 2923, 1709, 1610, 1586, 1150, 1069 cm^−1^; ^1^H-NMR (500 MHz, CDCl_3_-CD_3_OD) and ^13^C-NMR (125 MHz, CDCl_3_-CD_3_OD): see Table 1. HRESI–MS *m*/*z* 405.1300 [M + Na]^+^ (calcd. for C_22_H_22_O_6_Na^+^ *m*/*z* 405.1308).

### 4.5. Cylindricine A (***2***)

Yellow amorphous powder; IR (ν_max_) 3233, 2921, 1634, 1600, 1104 cm^−1^; ^1^H-NMR (500 MHz, CD_3_OD) and ^13^C-NMR (125 MHz, CD_3_OD): see Table 2. HRESI–MS *m*/*z* 371.1478 [M + H]^+^ (calcd. for C_21_H_23_O_6_^+^ *m*/*z* 371.1489).

### 4.6. Cylindricine B (***3***)

Yellow amorphous powder; IR (ν_max_) 3355, 2922, 1642, 1600 and 1087 cm^−1^; ^1^H-NMR (500 MHz, CD_3_OD) and ^13^C-NMR (125 MHz, CD_3_OD): see Table 2. HRESI–MS *m*/*z* 391.1140 [M + Na]^+^ (calcd. for C_21_H_20_O_6_Na^+^ *m*/*z* 391.1152).

### 4.7. Cylindracid A (***4***)

Brown oil; ${\left[\alpha \right]}_{D}^{20}$: +33.6 (*c* 0.17, CH_2_Cl_2_-MeOH 1:1); IR (ν_max_) 3377, 2977, 1707, 1663, 1060 cm^−1^; ^1^H-NMR (500 MHz, CD_3_OD) and ^13^C-NMR (125 MHz, CD_3_OD): see Table 1. HRESI–MS *m*/*z* 289.1407 [M + Na]^+^ (calcd. for C_15_H_22_O_4_Na^+^, *m*/*z* 289.1410).

### 4.8. Cylindracid B (***5***)

Brown oil; ${\left[\alpha \right]}_{D}^{20}$: +29.6 (*c* 0.167, CH_2_Cl_2_-MeOH 1:1); IR (ν_max_) 3365, 2922, 1642, 1600, 1504, 1439, 1312, 1088 cm^−1^; ^1^H-NMR (500 MHz, CD_3_OD) and ^13^C-NMR (125 MHz, CD_3_OD): see Table 1. HRESI–MS [M-H]^−^ at *m*/*z* 211.0985 (calcd. *m*/*z* 211.0975 for C_11_H_15_O_4_^−^).

### 4.9. Bioactivity: INT Colorimetric Assay for Minimal Inhibitory Concentration (MIC) and Minimum Bactericidal Concentration (MBC) Determinations

The bacterial growth inhibition capacity of the crude methanol extract of *Imperata cylindrica* and its isolated compounds was assessed on a panel of six multi-drug resistance (MDR) bacteria: *E. coli* (ATCC 8739, AG 102), *Enterobacter aerogenes* (ATCC 13048, EA27), and *Staphylococcus aureus* (ATCC 25923, MRSA1). The MIC and MBC determinations were performed using the rapid INT colorimetric assay as described by [44], with some modifications as previously described [45,46]. Briefly, 100 μL of Müller Hinton Broth (MHB) was introduced into each well of a 96-well microtiterplate, followed by introduction of 100 μL solution of samples (crude extract, isolated compounds or chloramphenicol as reference). DMSO was used to dissolve the samples, at a final concentration below 1%. Preliminary assays with DMSO 1% did not show any effect on the growth of the tested bacteria. Two-fold dilution series of samples were made and the tested bacterial concentration was 1.5 × 10^6^ colony forming unit (CFU)/mL. Plates were sealed with parafilm and incubated at 37 °C for 18 h. The negative control consisted of wells containing only MHB and inoculum, while the positive control was made up of MHB, inoculum, and a reference antibiotic (chloramphenicol). After 18 h incubation at 37 °C, the MIC of each sample was determined as the lowest sample concentration at which no bacterial growth was observed following the addition of 40 μL INT (0.2%). MBC was detected by adding 50 μL aliquots of the preparations, which did not show any growth after incubation during MIC assays, to 150 μL of MHB. These preparations were further incubated at 37 °C for 48 h. All the assays were performed in triplicate and repeated three times.

## 5. Conclusions

This study afforded five compounds unprecedented in nature, flavonoids (**1**–**3**) and C-15 sesquiterpenoid analogues (**4** and **5**). These findings enrich the chemotaxonomy of the plant, genus, and even family studied. The isolated compounds display considerable antibacterial activity against MDR strains. This study supports the uses of the plant in folk medicine to relieve urinary infections and might lead to an eventual standardization of a phytodrug based on both the chemical constituents and the biological features highlighted here amongst others.

## Figures and Tables

**Table 1:** ^1^H- (500 MHz) and ^13^C-NMR (125 MHz) data of 1 (CDCl_3_-CD_3_OD) and, 4 and 5 (CD_3_OD).

Position	1			4				5	
	*δ*_C_	*δ*_H_	HMBC	*δ*_C_	*δ*_H_	HMBC	*δ*_C_	*δ*_H_	HMBC
**1**				46.8	-	-	46.6	-	-
**2**	152.9	-	-	205.8	-	-	205.7	-	-
**2^1^**	109.7	6.90 s	3, 3′, 2′, 6′, 1′						
**3**	182.5	-	-	124.1	5.82 d (10.3)	1, 6, 4, 14	125.4	5.84 d (10.3)	6, 4
**3a**	105.5	-	-						
**4**	170.4	-	-	156.2	6.78 d (10.3)	3, 5, 7, 14	157.4	6.80 d (10.3)	3, 5
**5**	97.7	6.13 d (2.1)	4, 7, 8	71.9	-	-	71.4	-	-
**6**	169.2	-	-	49.6	2.86 m	8, 6, 4	51.3	2.87 m	6, 4, 10
**7**	90.2	6.38 d (2.1)	5, 6, 9	33.3	2.99 dd (15.9, 6.1) 2.88 dd (15.9, 6.1)	8, 6, 5, 4	31.5	2.63 dd (15.7, 6.3) 2.42 dd (15.7, 6.3)	8, 6, 5, 4 8, 6, 5, 4
**7a**	159.3	-	-						
**8**				203.4	-	-	177.5	-	-
**9**				137.5	-	-	24.7	1.11 s	3, 5, 4
**10**				140.9	6.80 d (3.4)	8, 9, 15	21.7	1.05s	3, 5, 4, 9
**11**				58.9	4.36 d (5.7)	8, 10, 9,15	23.1	1.32 s	1, 6, 5
**12**				25.0	1.01 s	3, 5, 4, 13			
**13**				21.8	1.07 s	3, 5, 4, 12			
**14**				23.0	1.34 s	1, 6, 5			
**15**				10.9	1.75 s	8, 10, 9			
**1′**	124.0	-	-						
**2′**	138.3	-	-						
**3′**	148.1	-	-						
**4′**	147.0	-	-						
**5′**	115.5	6.83 d (8.2)	2, 4′, 2′, 1′						
**6′**	129.5	7.93 d (8.2)	2, 4′, 2′, 10						
**1″**	26.3	3.55 d (6.7)	4″, 5″, 4′, 2′						
**2″**	124.7	5.09 m	4″, 5″						
**3″**	132.9	-	-						
**4″**	25.9	1.70 s	3″, 2″, 5″						
**5″**	18.2	1.90 s	3″, 2″, 4″						
**3ʹ-OMe**	61.2	3.78 s	3′						
**6-OMe**	56.5	3.90 s	6						

**Table 2:** ^1^H- (500 MHz) and ^13^C-NMR (125 MHz) data of 2 and 3 in CD_3_OD.

Position	2			3		
	*δ*_C_	*δ*_H_	HMBC	*δ*_C_	*δ*_H_	HMBC
**2**	80.5	5.23 dd (12.5, 3.1)	4, 8a, 1′, 6′, 2′	146.1	-	-
**3**	44.1	3.04 dd (17.0, 12.5) 2.70 dd (17.1, 3.1)	4, 1′, 2, 4, 1′, 5a	113.3	6.55 s	4, 2, 5′
**4**	197.7	-	-	183.9	-	-
**4a**	103.0	-	-	104.4	-	-
**5**	162.4	-	-	166.5	-	-
**6**	95.2	5.95 s	4, 5, 7, 8, 5a	91.5	6. 28 s	8, 5a, 5, 7
**7**	166.0	-	-	167.7	-	-
**8**	105.2	-	-	107.3	-	-
**8a**	162.1	-	-	156.6	-	-
**1′**	130.9	-	-	124.7	-	-
**2′**	111.7	6.78 d (2.2)	3′, 4′, 6′, 2, 1″	116.1	7.33 d (2.2)	3′
**3′**	145.8	-	-	147.0	-	-
**4′**	144.4	-	-	146.7	-	-
**5′**	129.5	-	-	129.8	-	-
**6′**	119.7	6.69 d (2.2)	4′, 2′, 2, 1″	126.3	7.13 d (2.2)	1″, 2, 2′, 3′
**1″**	29.3	3.32 m	4′, 3″, 5′, 2″, 6′	29.9	3.34 m	3″, 5′, 6′, 3′
**2″**	123.7	5.33 m	4″, 3″	123.6	5.39 d (2.2)	5″, 4″, 1″
**3″**	132.8	-	-	133.4	-	-
**4″**	25.6	1.73 s	3″, 2″	26.1	1.80 s	3″, 2″, 5″
**5″**	17.2	1.74 s	3″, 2″	17.9	1.78 s	3″, 2″, 4″
**1‴**	6.9	1.96 s	7, 8a, 8	7.4	2.3 s	7, 8a, 15

**Table 3:** MIC and MBC values (μg/mL) of crude extract and isolated compounds.

Bacterial Strains	Tested Samples															
	Crude Extract		1		2		3		4		5		6		CHL	
	MIC	MBC	MIC	MBC	MIC	MBC	MIC	MBC	MIC	MBC	MIC	MBC	MIC	MBC	MIC	MBC
*E. coli*																
ATCC 8739	512	-	128	-	128	-	128	-	1024	-	512	-	128	-	64	512
AG102	1024	-	1024	-	128	-	256	-	512	-	256	-	64	-	64	512
*E. aerogenes*																
ATCC 13048	256	-	1024	-	128	-	64	-	512	-	64	-	128	-	128	128
EA 27	512	-	512	-	128	-	128	-	512	-	128	-	128	-	64	256
*S. aureus*																
ATCC 25923	128	-	1024	-	32	-	128	-	512	-	256	-	32	-	32	-
MRSA 1	256	-	512	-	16	-	64	-	512	-	128	-	128	-	64	-

*E. coli: Escherichia coli, E aerogenes: Enterobacter aerogenes, S. aureus: Staphylococcus aureus*, MIC: minimal inhibitory concentration, MBC: minimal bactericidal concentration, CHL: chloramphenicol, (-): not determined.

## Data Availability

The raw data supporting the conclusions of this article will be made available by the authors, without undue reservation, to any qualified researcher.

## Supplementary Materials

The following are available online. Mass, IR, and NMR spectra (Figures S1–S38) of compounds **1**–**5**.

## Sample Availability

Samples of the compounds are not available from the authors.
