# Supplementary material for: Prenylated Flavonoids and C-15 Isoprenoid Analogues with Antibacterial Properties from the Whole Plant of Imperata cylindrica (L.) Raeusch (Gramineae)
Source: Molecules. 2021 Aug 4;26(16):4717. doi: 10.3390/molecules26164717 (PMC8401516; doi:10.3390/molecules26164717)
Supplement: Supplementary file 1 [file molecules-26-04717-s001.zip › molecules-1298805-supplementary.pdf]

Prenylated flavonoids and C-15 isoprenoid analogues with antibacterial properties from the whole plant of Imperata cylindrica (l.) Raeusch (Gramineae)

Romeo D. Tadjouate Nago[1], Paul Nayim[2], Armelle T. Mbaveng[2], James D. Simo Mpetga[1,*], Gabin T. Mbahbou Bitchagno[1,3,*], Badawe Garandi[2], Pierre Tane[†,1], Bruno N. Lenta[4], Norbert Sewald[3,*], Mathieu Tene[1], Victor Kuete[2], Augustin Silvere Ngouela[1]

1 Department of Chemistry, Faculty of Science, University of Dschang, P.O. Box 67, Dschang, Cameroon
2 Department of Biochemistry, Faculty of Science, University of Dschang, P.O. Box 67, Dschang, Cameroon
3 Organic and Bioorganic Chemistry, Department of Chemistry, Bielefeld University, D-33501, Bielefeld, Germany
4 Department of Chemistry, Higher Teacher Training College, University of Yaounde I, P. O. Box 47, Yaounde, Cameroon
* Correspondence: norbert.sewald@uni-bielefeld.de, gabin1256@gmail.com and james.mpetga@univ-dschang.org
† The manuscript is dedicated to the memory of Prof. Dr. Pierre Tane, who passed away on July 29, 2019, for his valuable contribution to natural products chemistry.

Spectroscopic data

- Cylindraucine (**1**), Nago et al. 2021

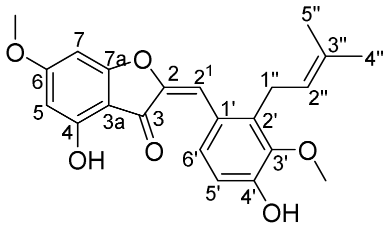

Yellowish powder, *m/z* 382.1, $C_{22}H_{22}O_6$; ^{1}H NMR (MeOD, 500 MHz): δ_H 6.90 (1H, s, H-2^1), 6.13 (1H, d, J = 2.1 Hz, H-5), 6.38 (1H, d, J = 2.1 Hz, H-7), 6.83 (1H, d, J = 8.2 Hz, H-5'), 7.93 (1H, d, J = 8.2 Hz, H-6'), 3.55 (2H, d, J = 6.7 Hz, H-1''), 5.09 (1H, m, H-2''), 1.70 (3H, s, H-4''), 1.90 (3H, s, H-5''), 3.78 (3H, s, MeO-3'), 3.90 (3H, s, MeO-6)
^{13}C NMR (MeOD, 125 MHz): δ_C 152.9 (C-2), 109.7 (C-2^1), 182.5 (C-3), 105.5 (C-3a), 170.4 (C-4), 97.7 (C-5), 169.2 (C-6), 90.2 (C-7), 159.3 (C-7a), 124.0 (C-1'),138.3 (C-2'), 148.1 (C-3'), 147.0 (C-4'), 115.5 (C-5'), 129.5 (C-6'), 26.3 (C-1''), 124.7 (C-2''), 132.9 (C-3''), 25.9 (C-4''), 18.2 (C-5''), 61.2 (MeO-3'), 56.5 (MeO-6).

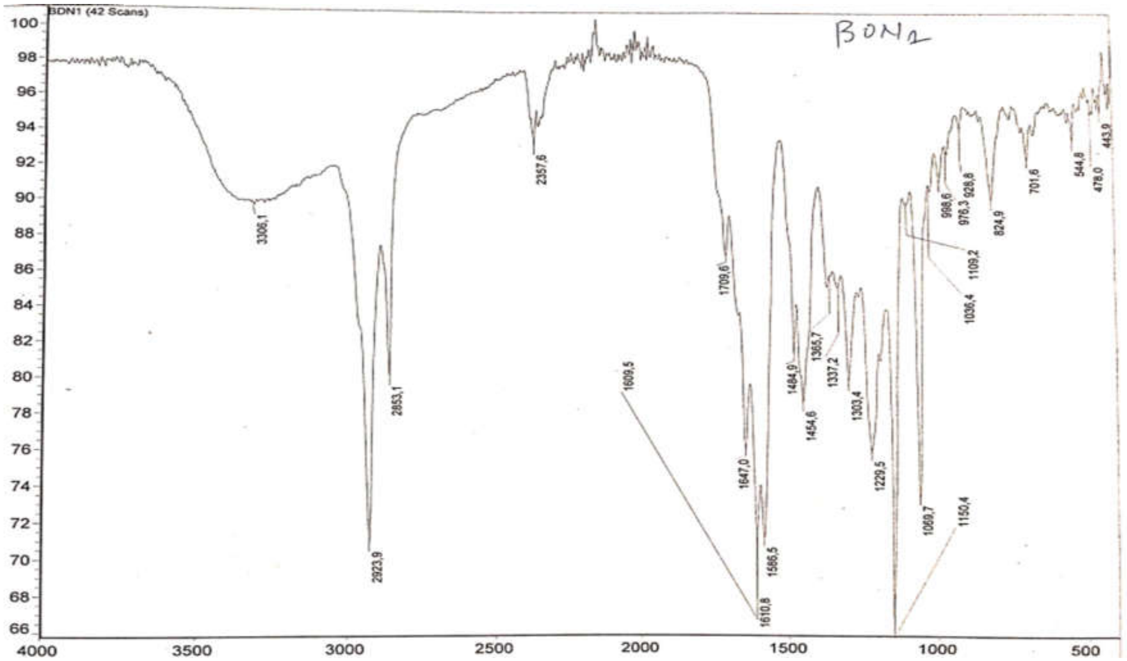

Figure **S1**: IR spectrum of **1**

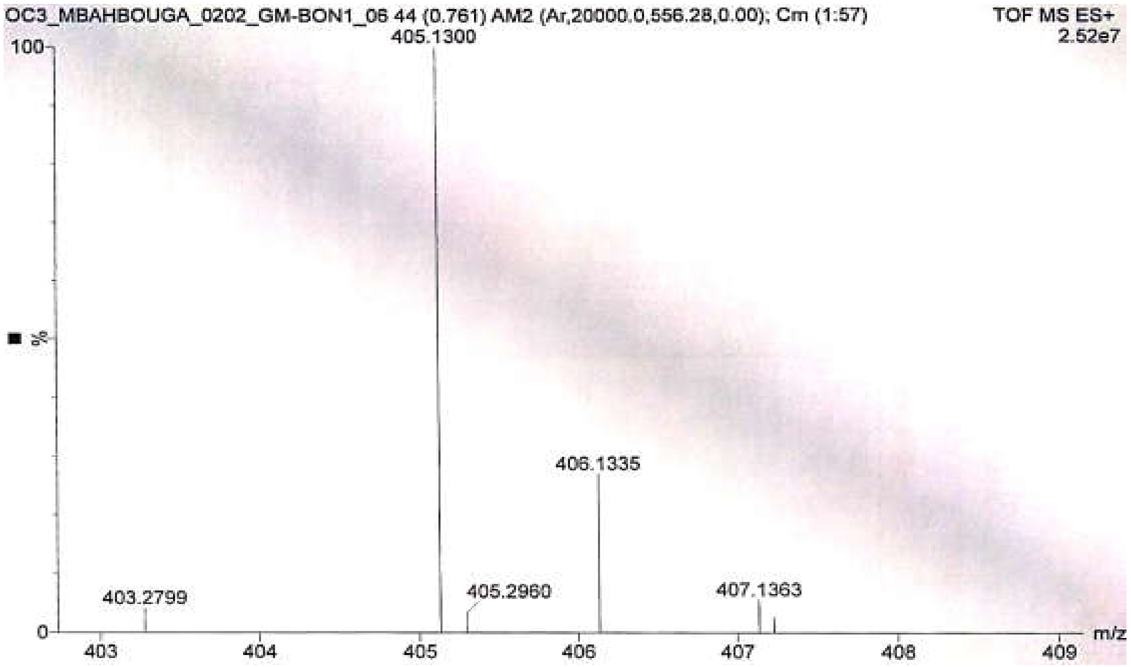

Figure **S2**: **HRESI-MS** spectrum of **1**

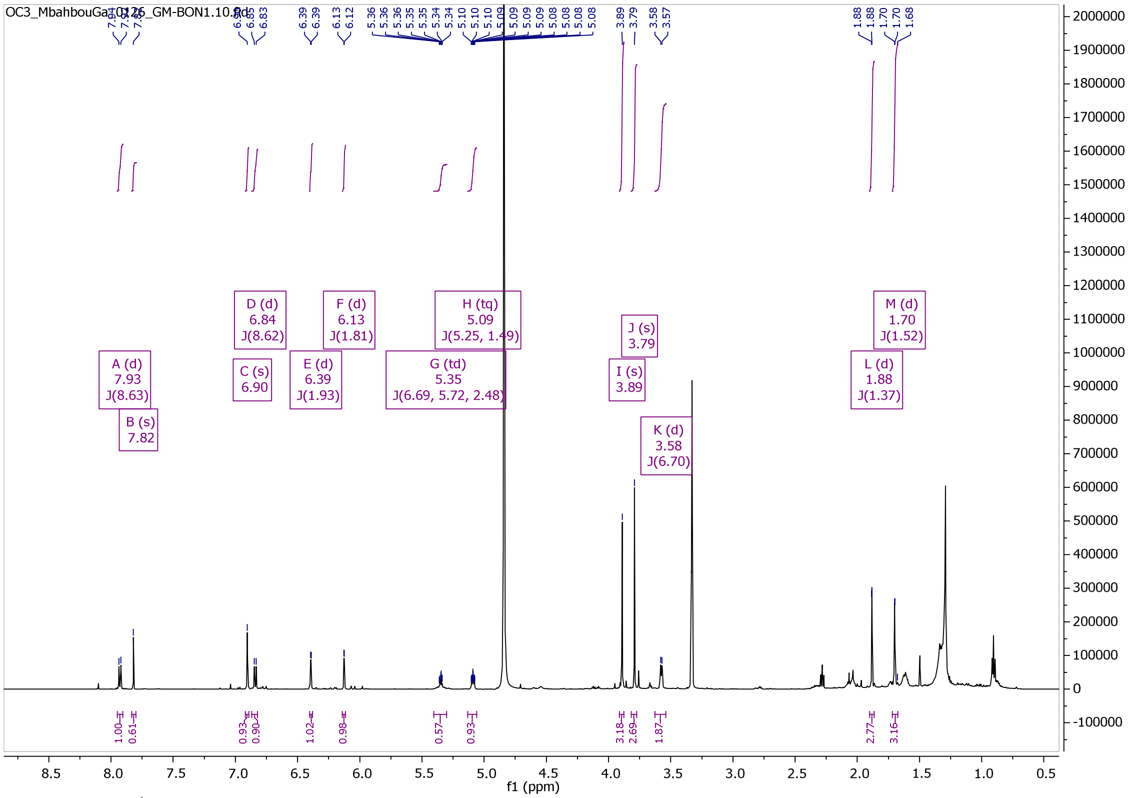

Figure S3. ^{1}H NMR spectrum of compound **1**

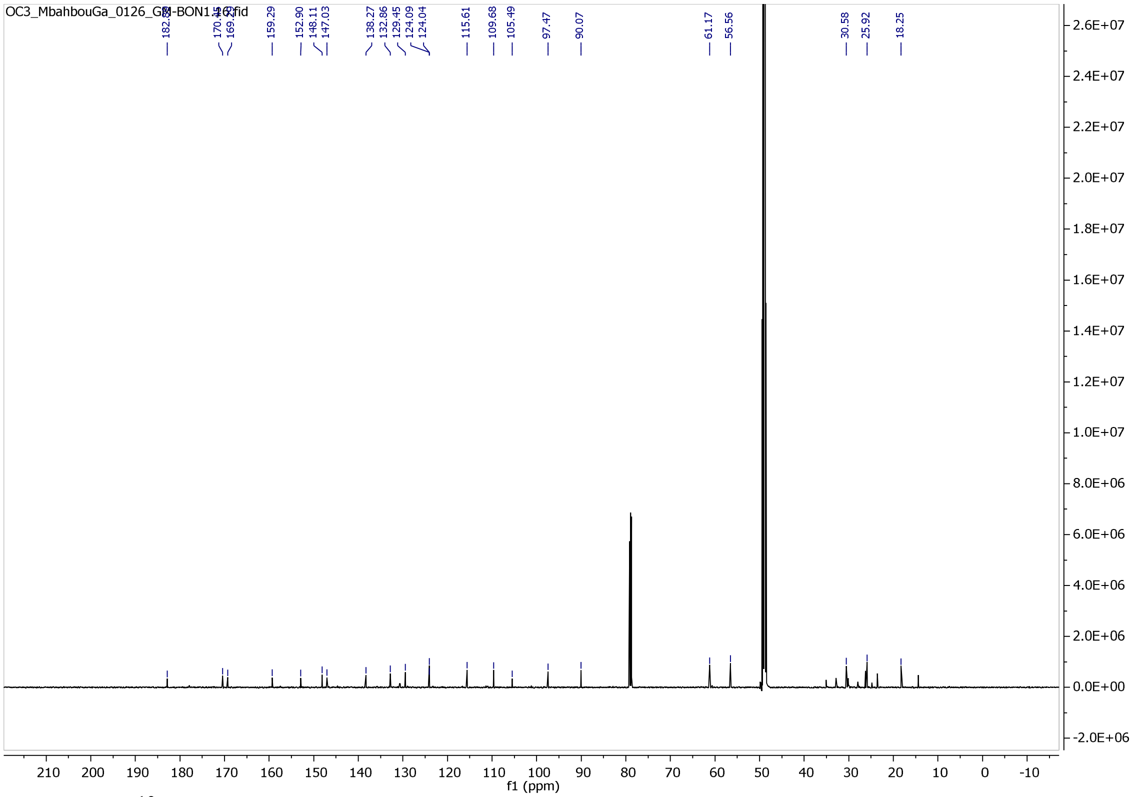

Figure S4. ^{13}C NMR spectrum of compound **1**

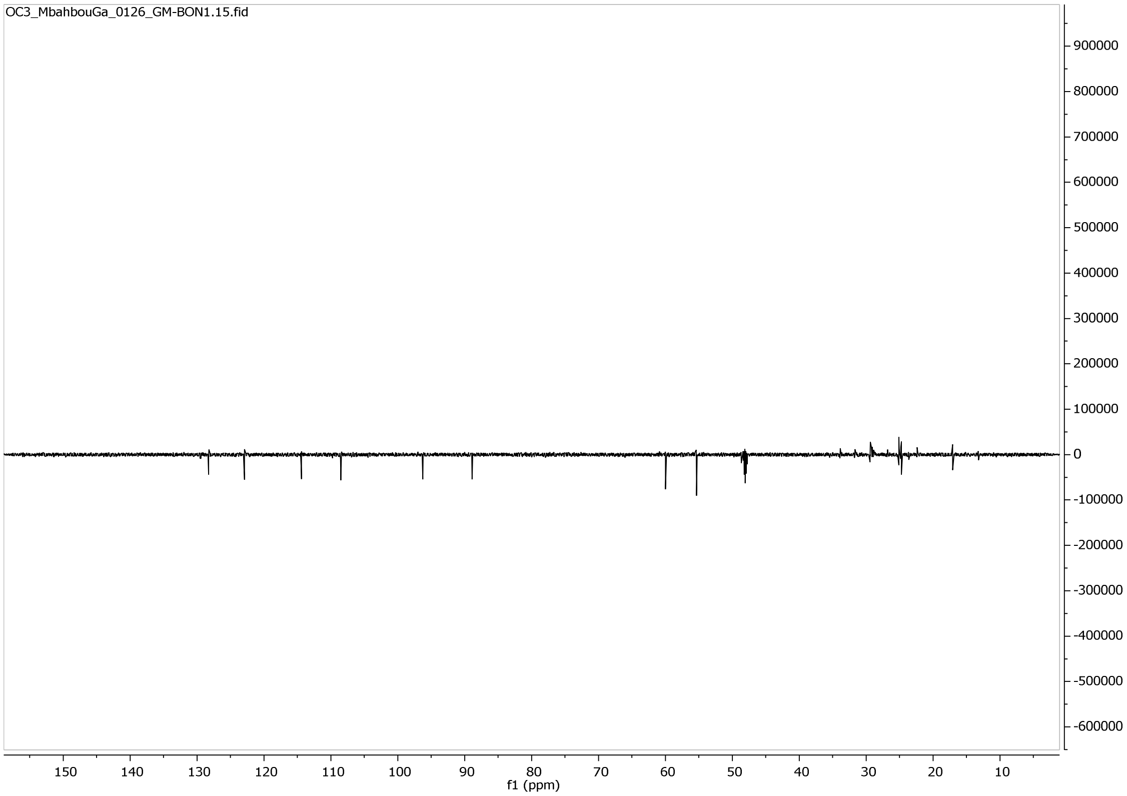

Figure S5. DEPT135 spectrum of compound **1**

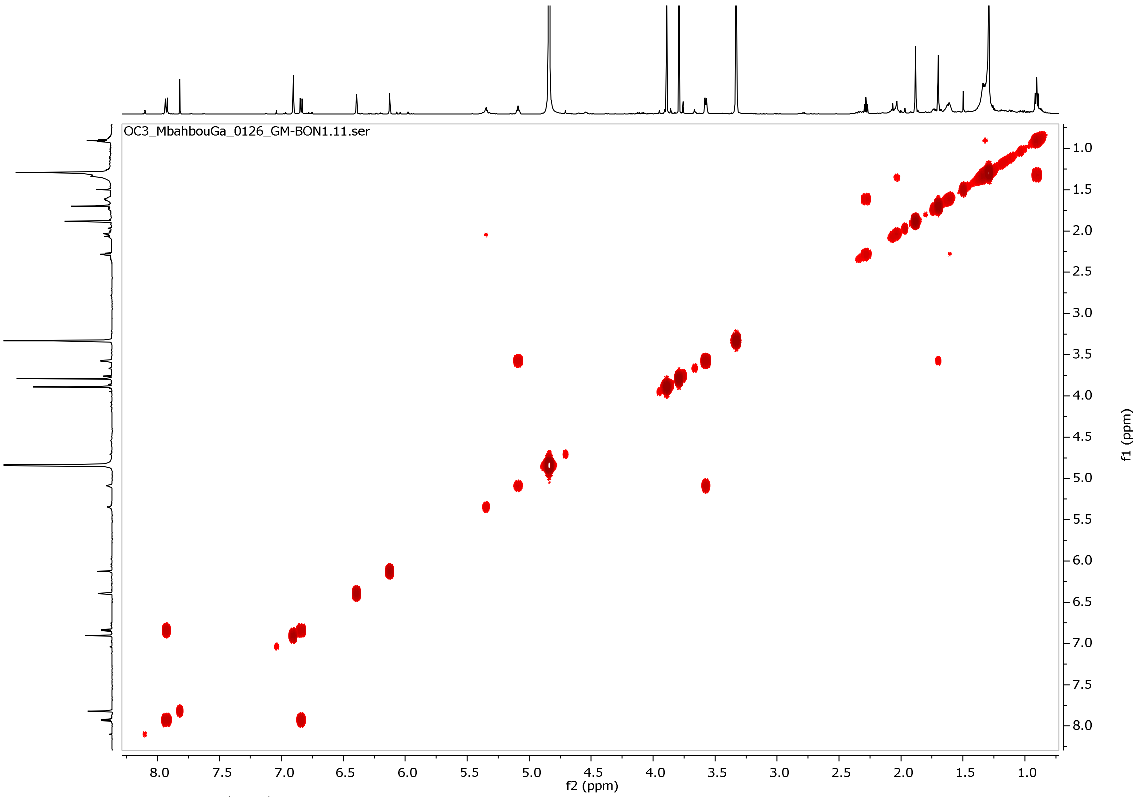

Figure S6. COSY $^{1}H,^{1}H$ spectrum of compound **1**

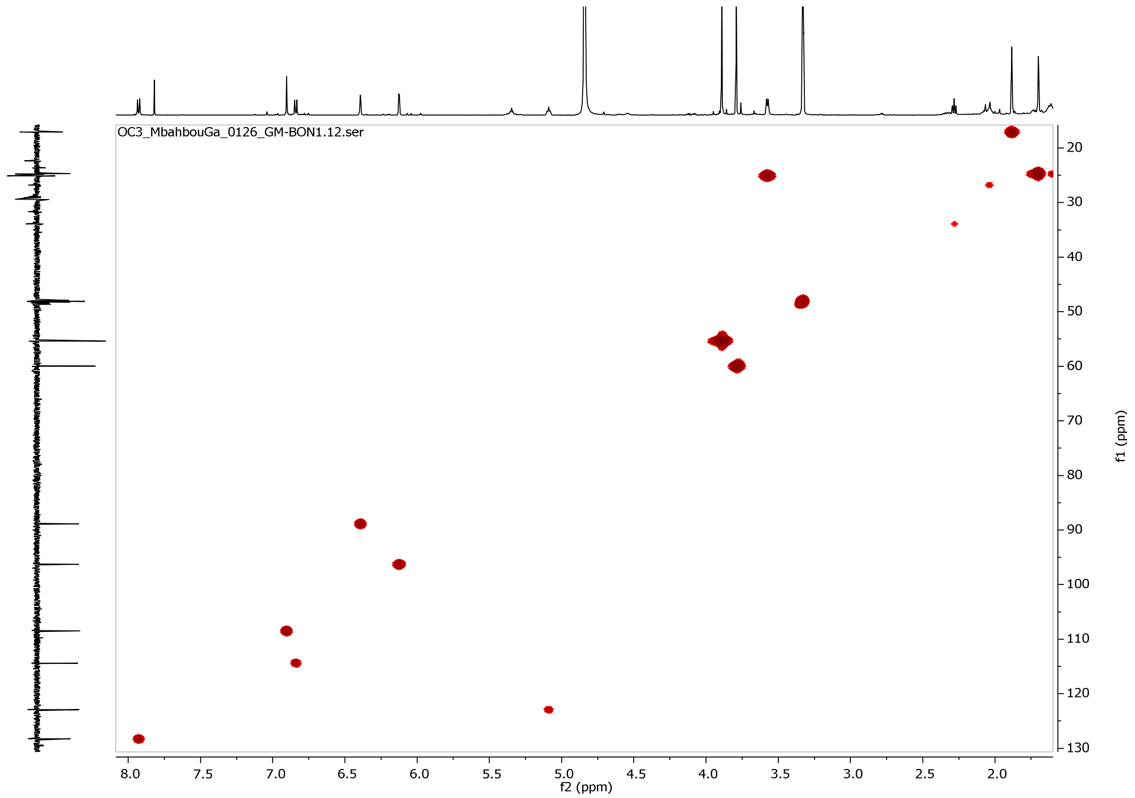

Figure S7. HSQC spectrum of compound **1**

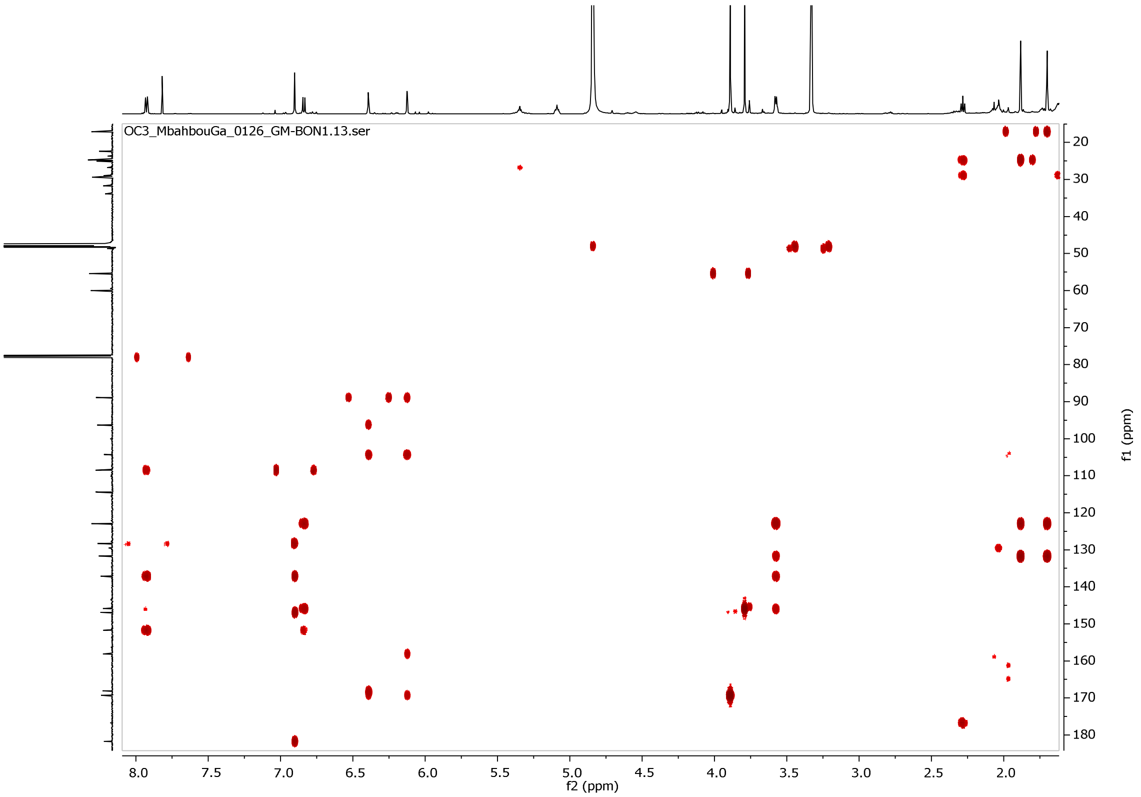

Figure S8. HMBC spectrum of compound **1**

- Cylindricine A (**2**), Nago et al. 2021

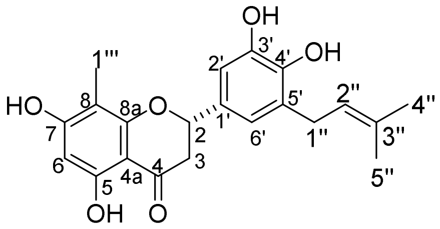

Yellowish powder, *m/z* 370.1, $C_{21}H_{22}O_6$; ^{1}H NMR (MeOD, 500 MHz): δ_H 5.23 (1H, dd J = 12.5 and 3.1 Hz, H-2), 3.04 (1H, dd J = 17.0 and 12.5 Hz, H-3a), 2.70 (1H, dd J = 17.0 and 3.1 Hz, H-3b), 5.95 (1H, s, H-6), 6.78 (1H, d J = 2.2 Hz, H-2'), 6.69 (1H, d J = 2.2 Hz, H-6'), 3.32 (2H, m, H-1''), 5.33 (1H, m, H-2''), 1.73 (3H, s, H-4''), 1.74 (3H, s, H-5''), 1.96 (3H, s, H-1''')

^{13}C NMR (MeOD, 125 MHz): δ_C 80.5 (C-2), 44.1 (C-3), 197.7 (C-4), 103.0 (C-4a), 162.4 (C-5), 95.2 (C-6), 166.0 (C-7), 105.2 (C-8), 162.1 (C-8a), 130.9 (C-1'), 111.7 (C-2'), 145.8 (C-3'), 144.4 (C-4'), 129.5 (C-5'), 119.7 (C-6'), 29.3 (C-1''), 123.7 (C-2''), 132.8 (C-3''), 25.6 (C-4''), 17.2 (C-5''), 6.9 (C-1''')

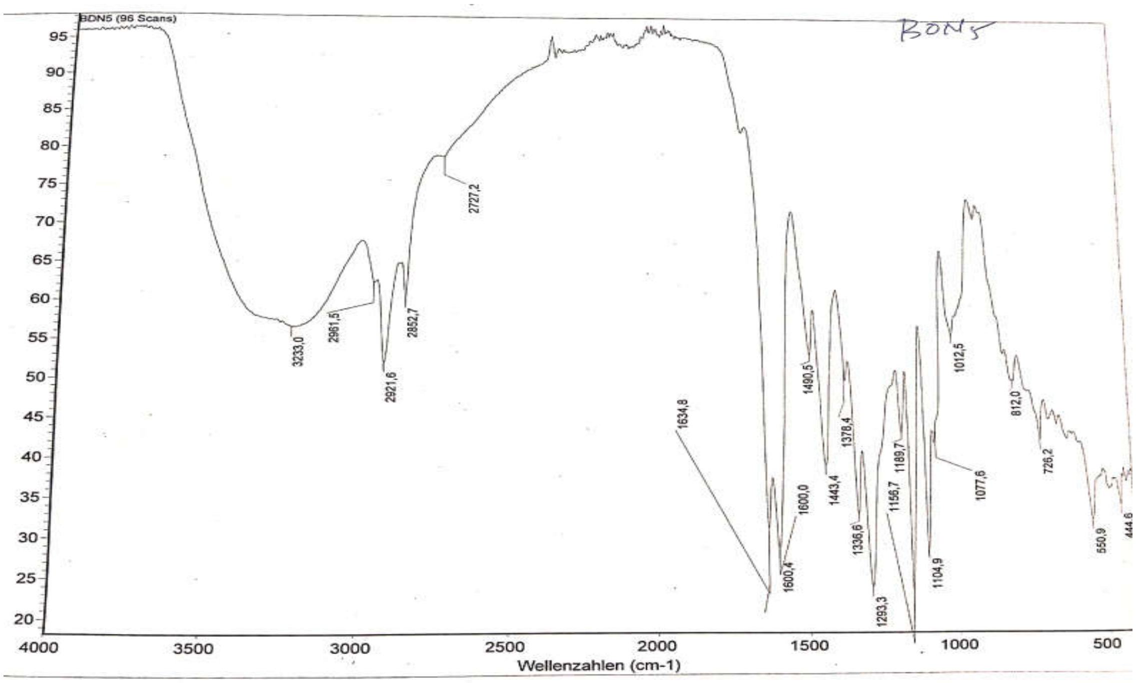

Figure **S9**: IR spectrum of **2**

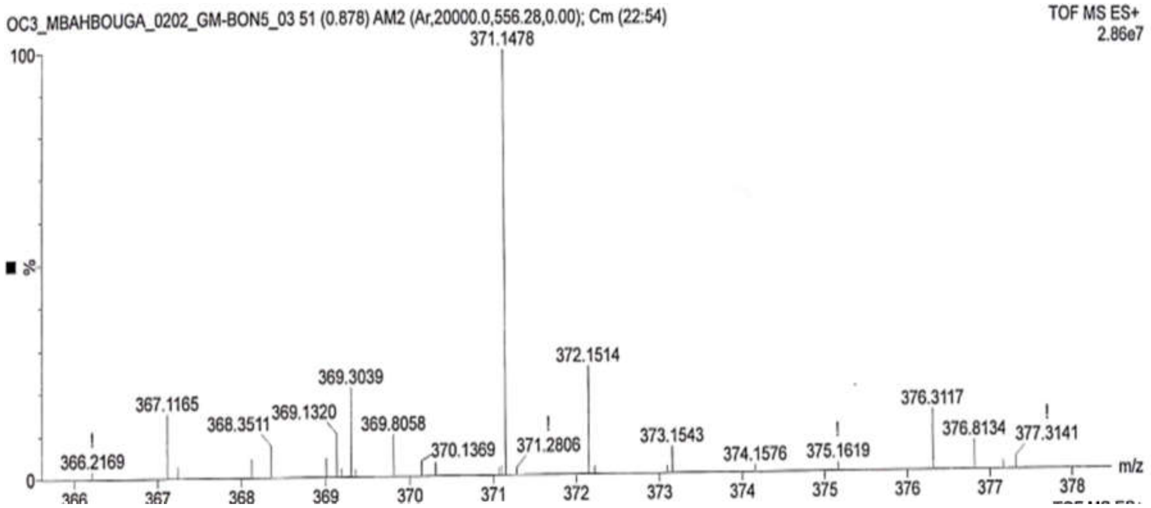

Figure **S10**: **HRESI-MS** spectrum of **2**

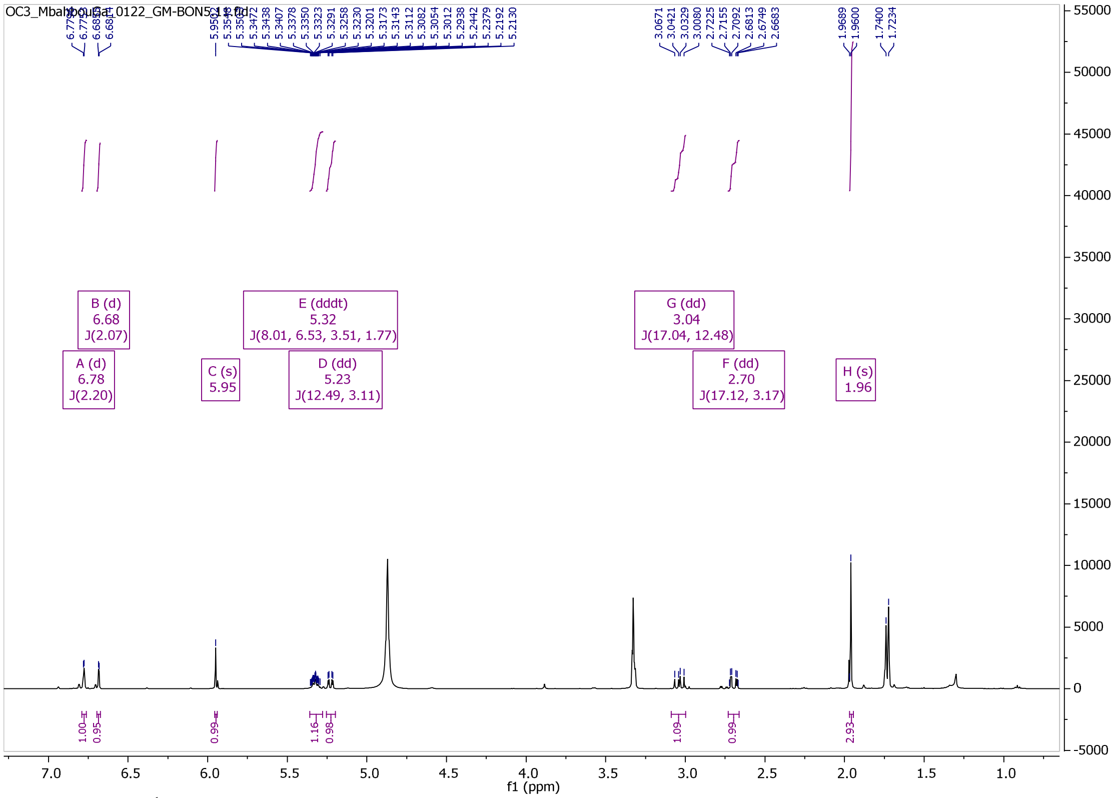

Figure S11. ^{1}H NMR spectrum of compound **2**

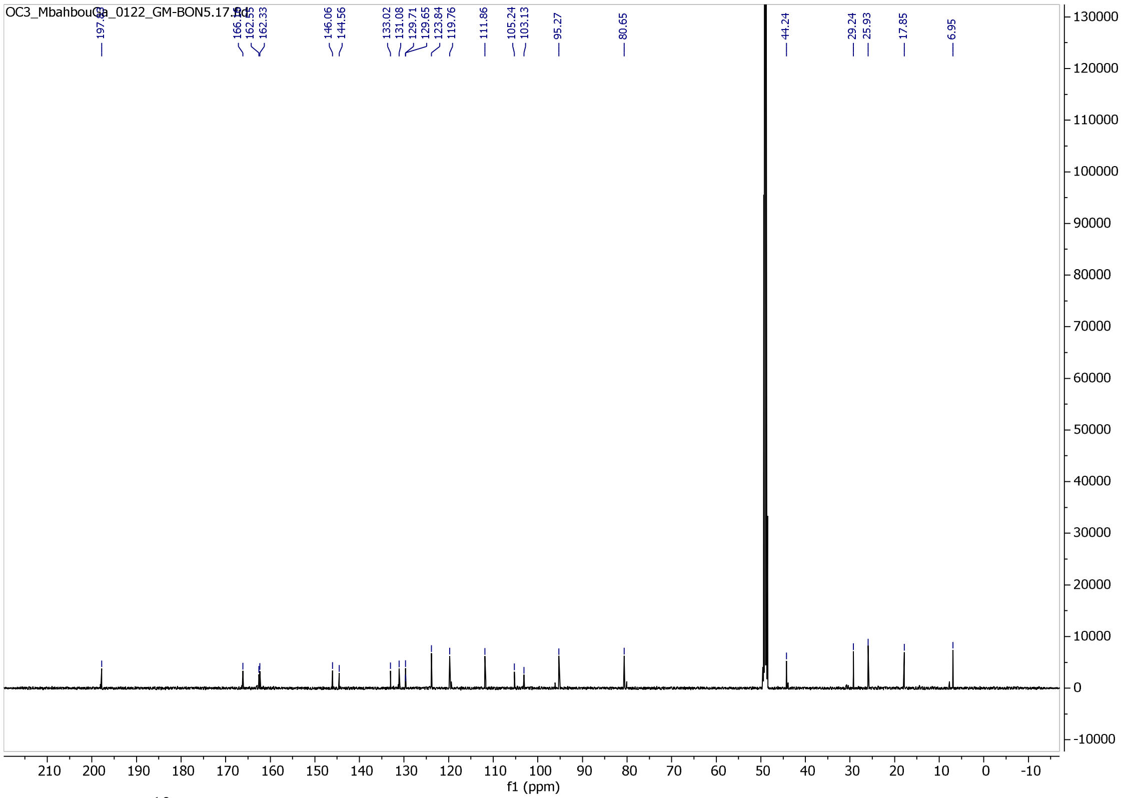

Figure S12. ^{13}C NMR spectrum of compound **2**

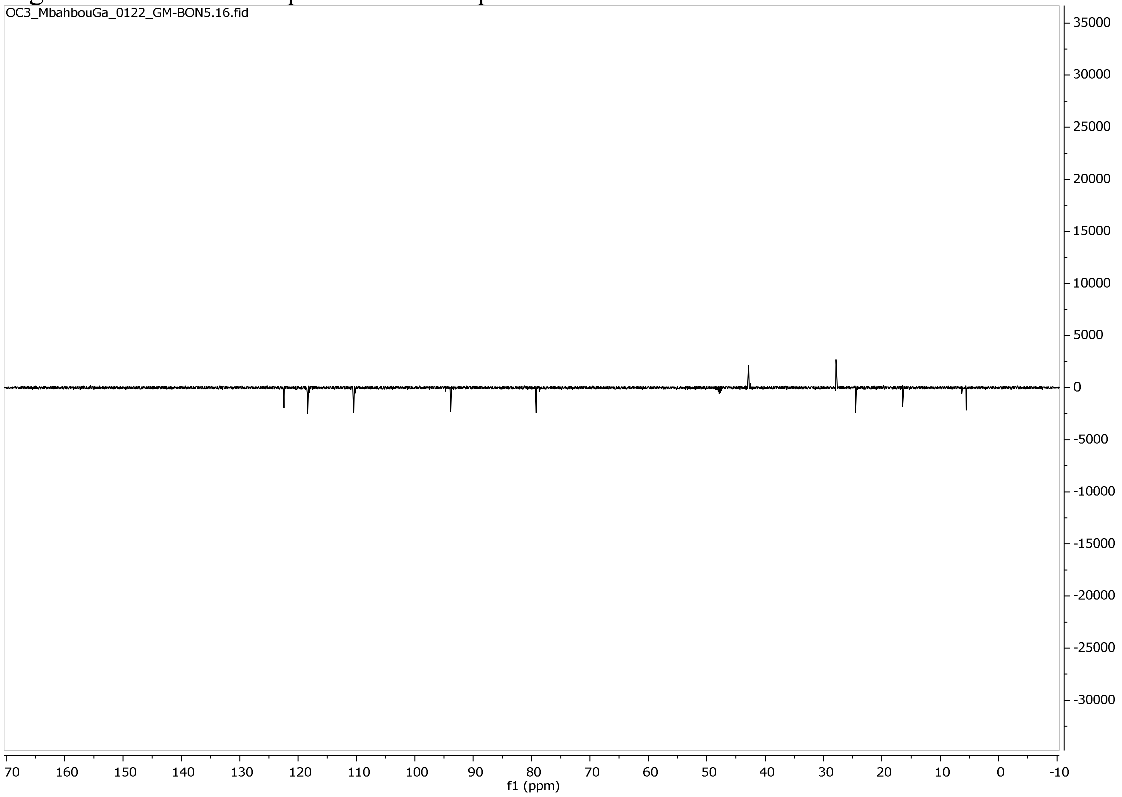

Figure S13. DEPT135 spectrum of compound **2**

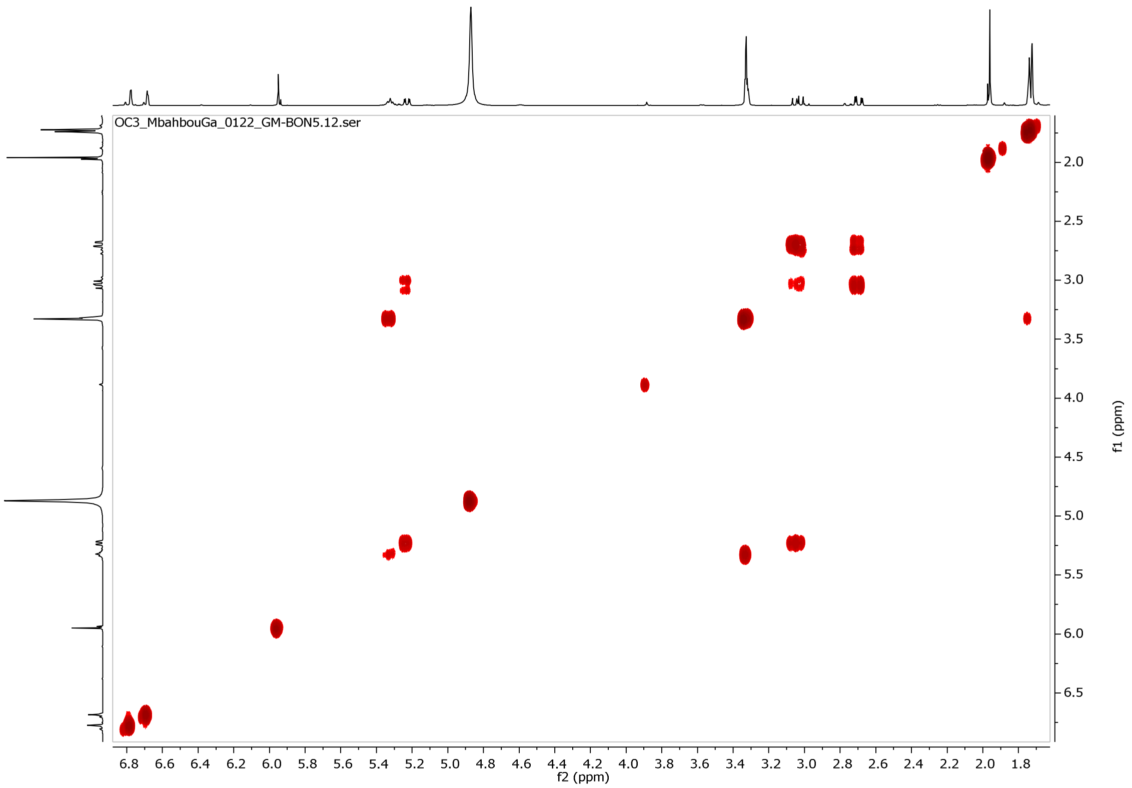

Figure S14. COSY spectrum of compound **2**

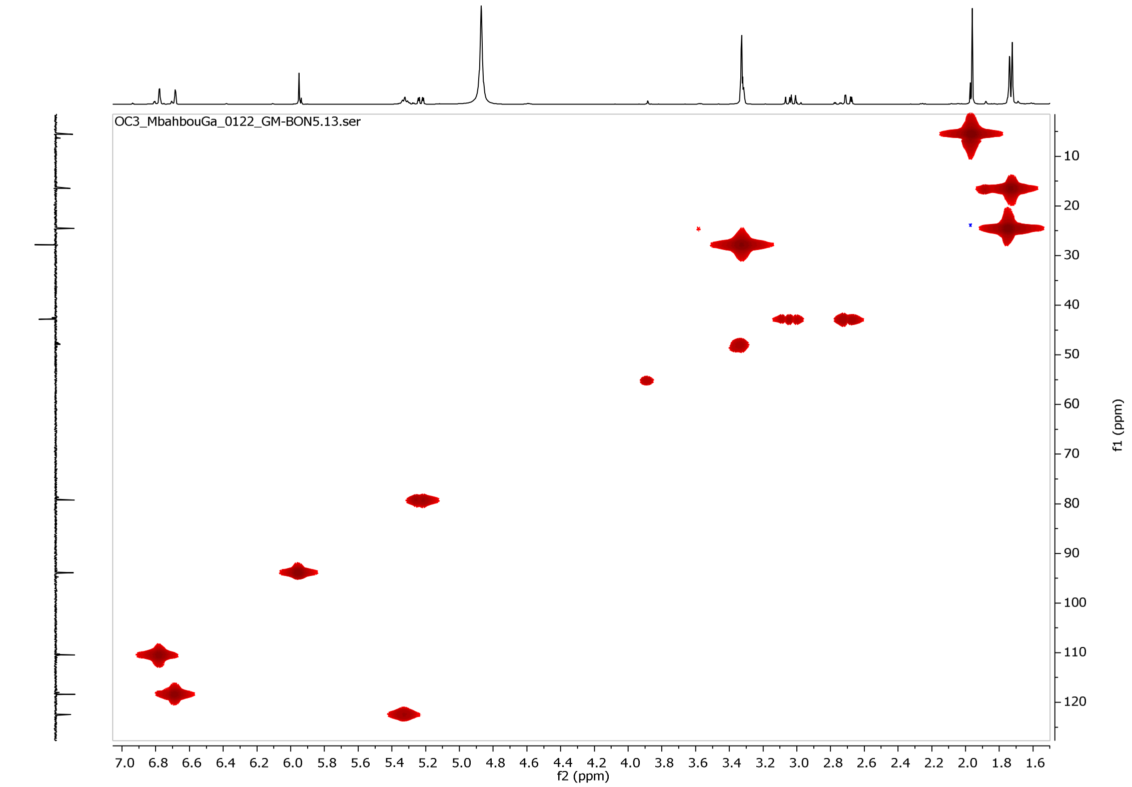

Figure S15. HSQC spectrum of compound **2**

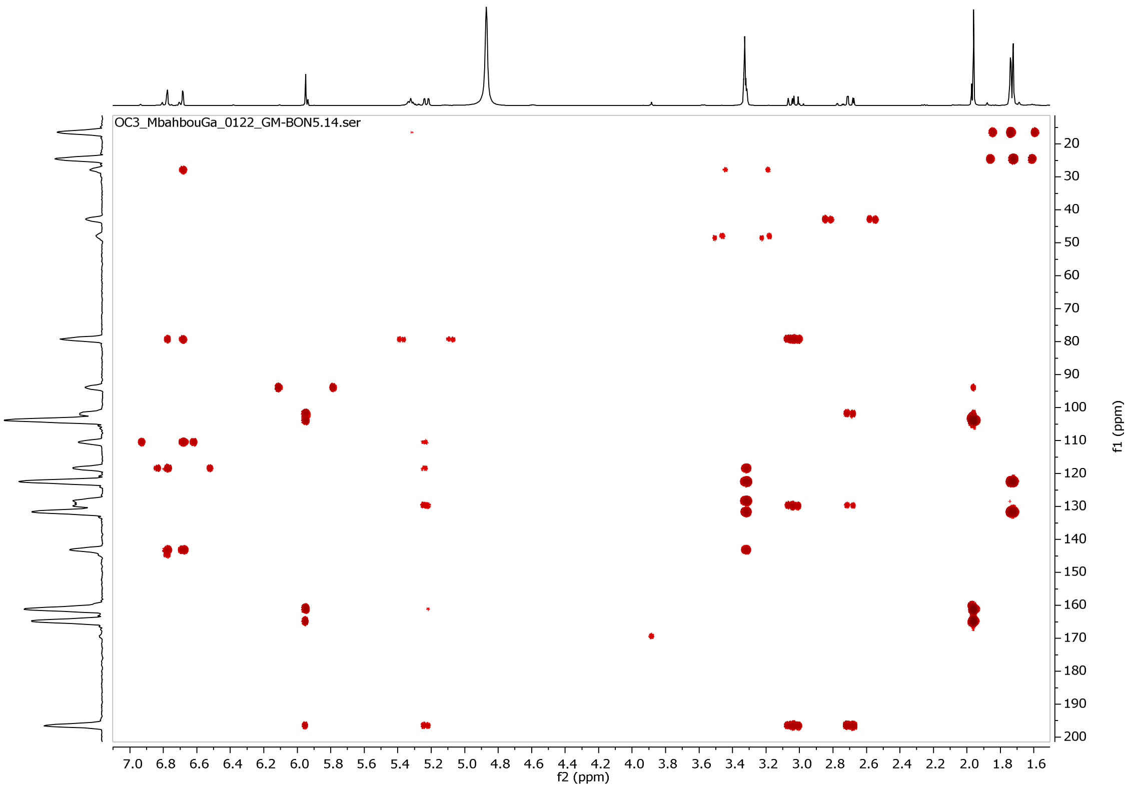

Figure S16. HMBC spectrum of compound **2**

- Cylindricine B (**3**), Nago et al. 2021

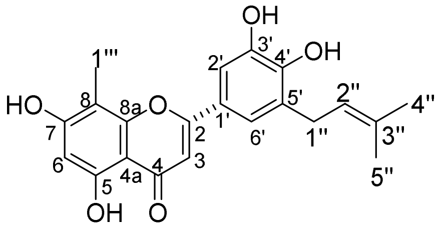

Yellowish powder, *m/z* 368.1, $C_{21}H_{20}O_6$; ^{1}H NMR (MeOD, 500 MHz): δ_H 6.55 (1H, s, H-3), 6. 28 (1H, s, H-6), 7.33 (1H, d, J = 2.2 Hz, H-2'), 7.13 (1H, d, J = 2.2 Hz, H-6'), 3.34 (2H, m, H-1''), 5.39 (1H, d, J = 2.2 Hz, H-2''), 1.80 (3H, s, H-4''), 1.78 (3H, s, H-5''), 2.3 (3H, s, H-1''')

^{13}C NMR (MeOD, 125 MHz): δ_C 146.1 (C-2), 113.3 (C-3), 183.9 (C-4), 104.4 (C-4a), 166.5 (C-5), 91.5 (C-6), 167.7 (C-7), 107.3 (C-8), 156.6 (C-8a), 124.7 (C-1'), 116.1 (C-2'), 147.0 (C-3'), 146.7 (C-4'), 129.8 (C-5'), 126.3 (C-6'), 29.9 (C-1''), 123.6 (C-2''), 133.4 (C-3''), 26.1 (C-4''), 17.9 (C-5''), 7.4 (C-1''')

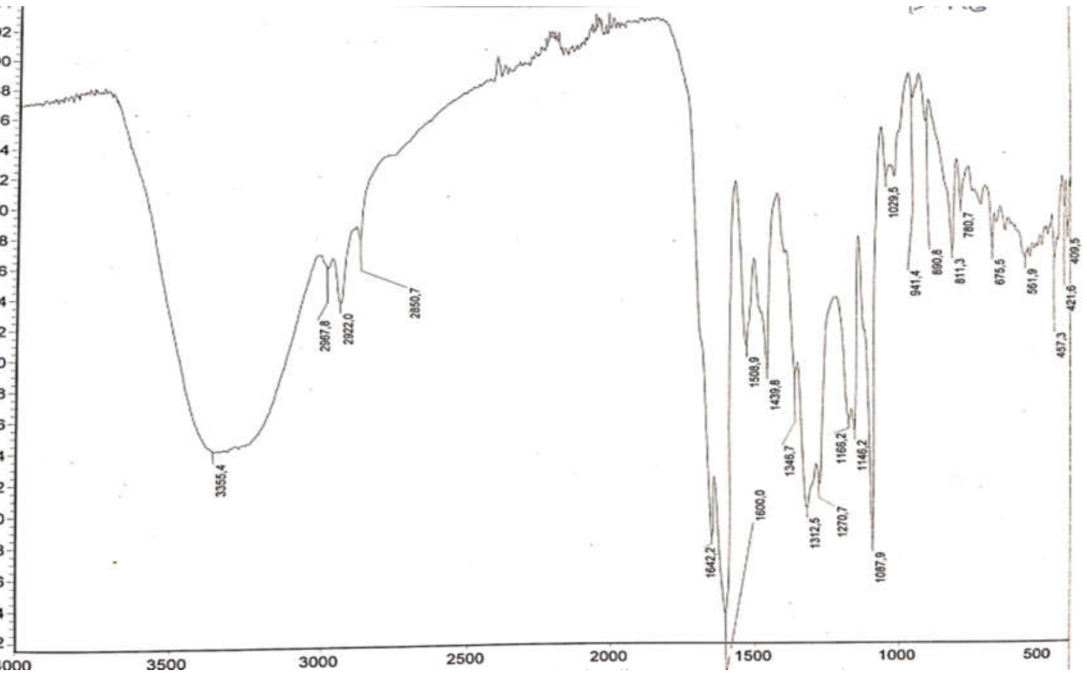

Figure **S17**: IR spectrum of **3**

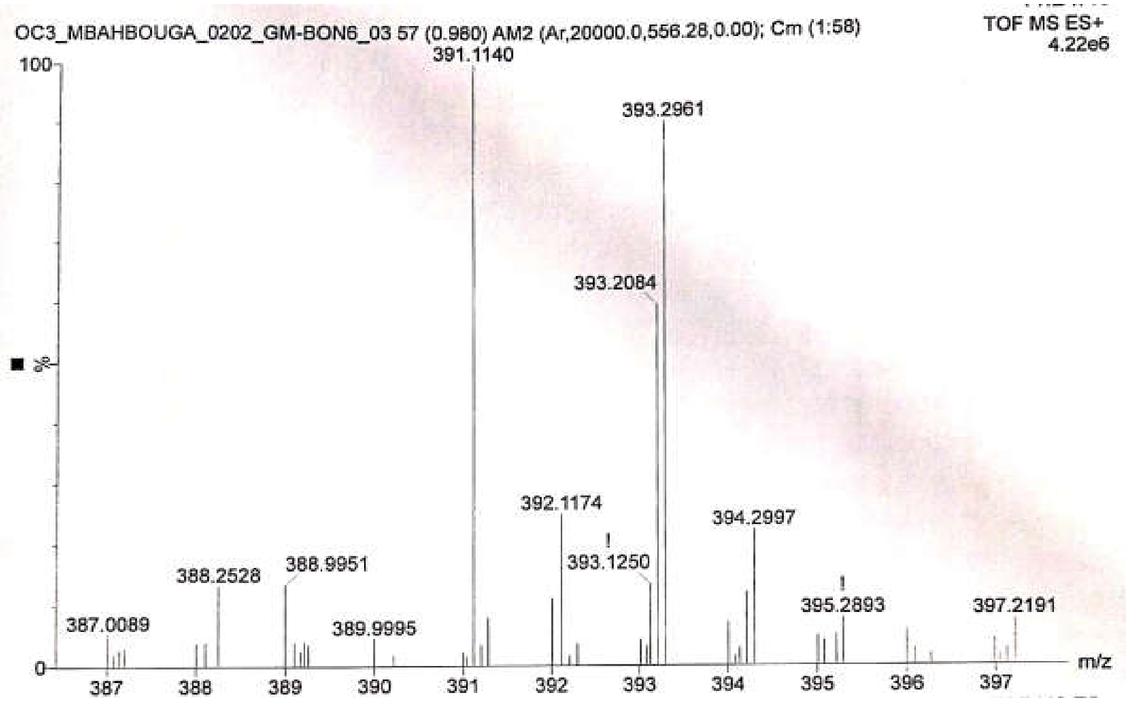

Figure **S18**: **HRESI-MS** spectrum of **3**

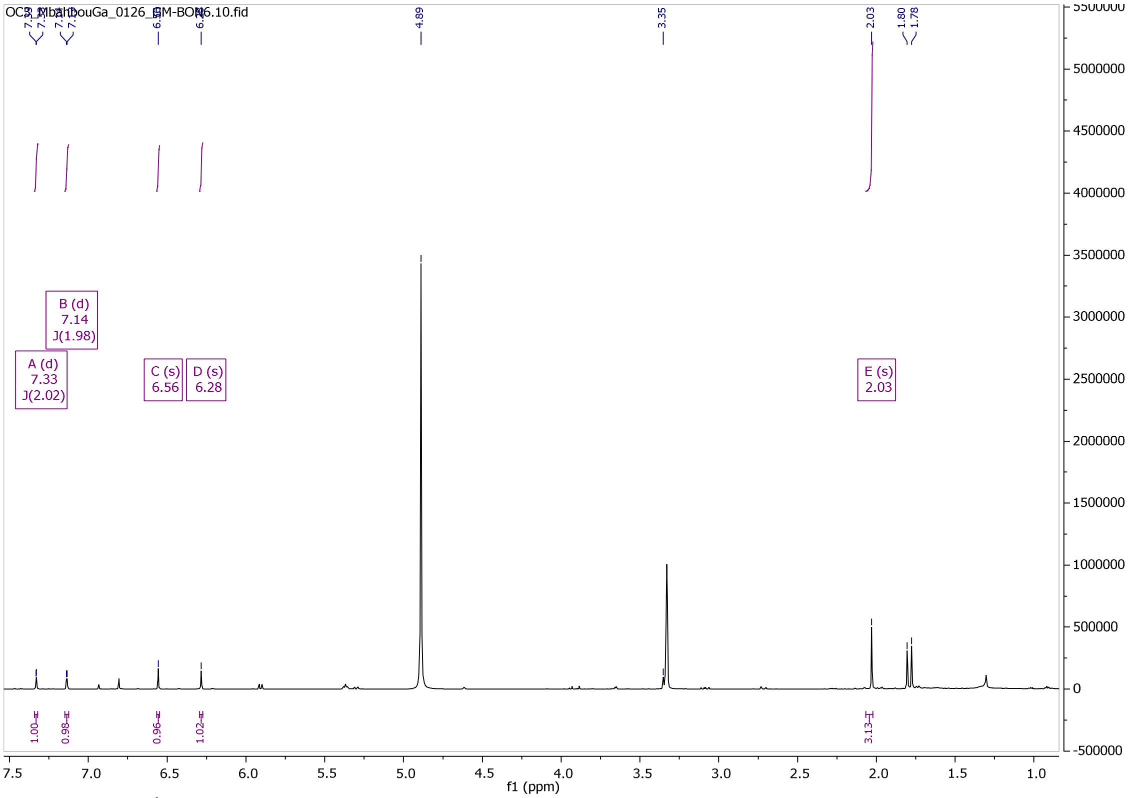

Figure S19. ^{1}H NMR spectrum of compound **3**

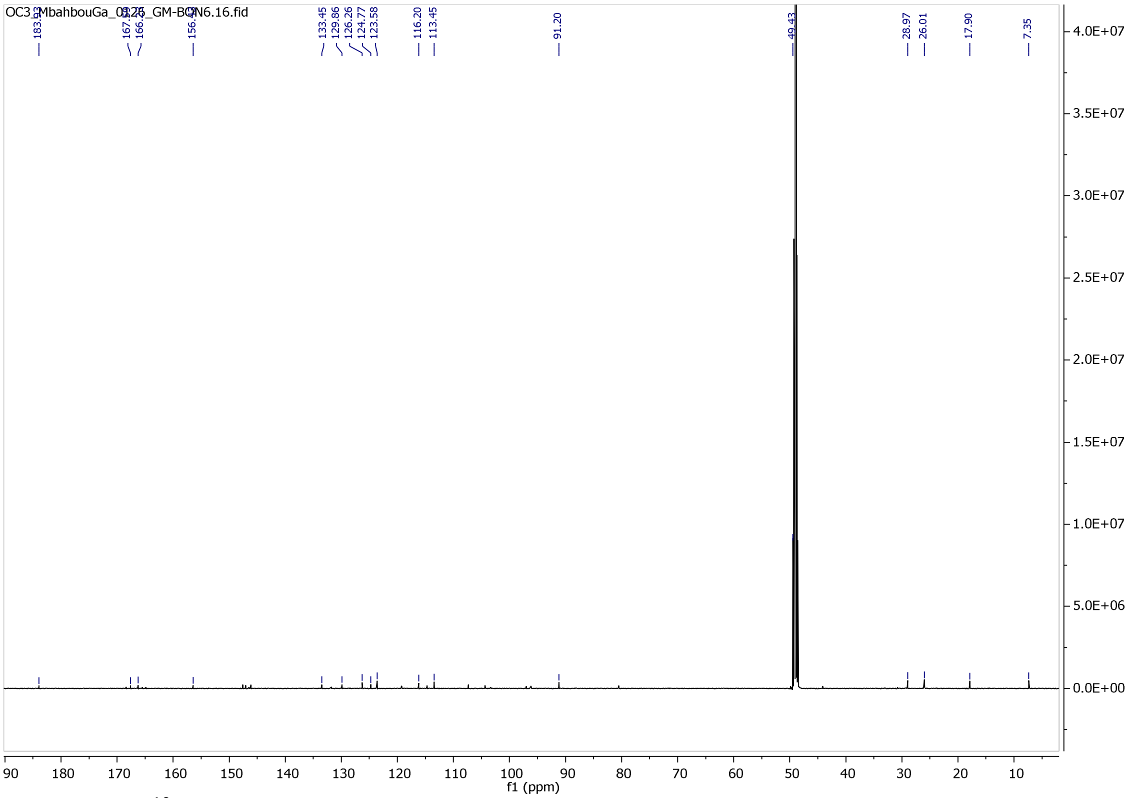

Figure S20. ^{13}C NMR spectrum of compound **3**

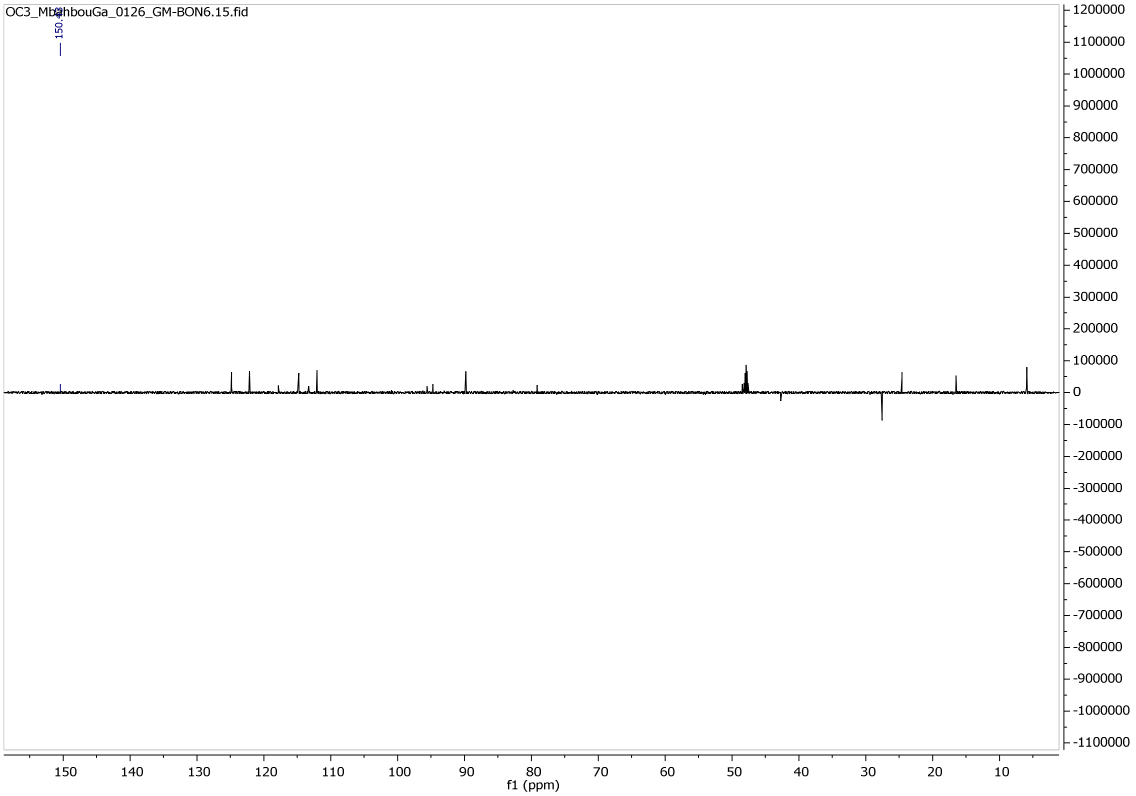

Figure S21. DEPT135 spectrum of compound **3**

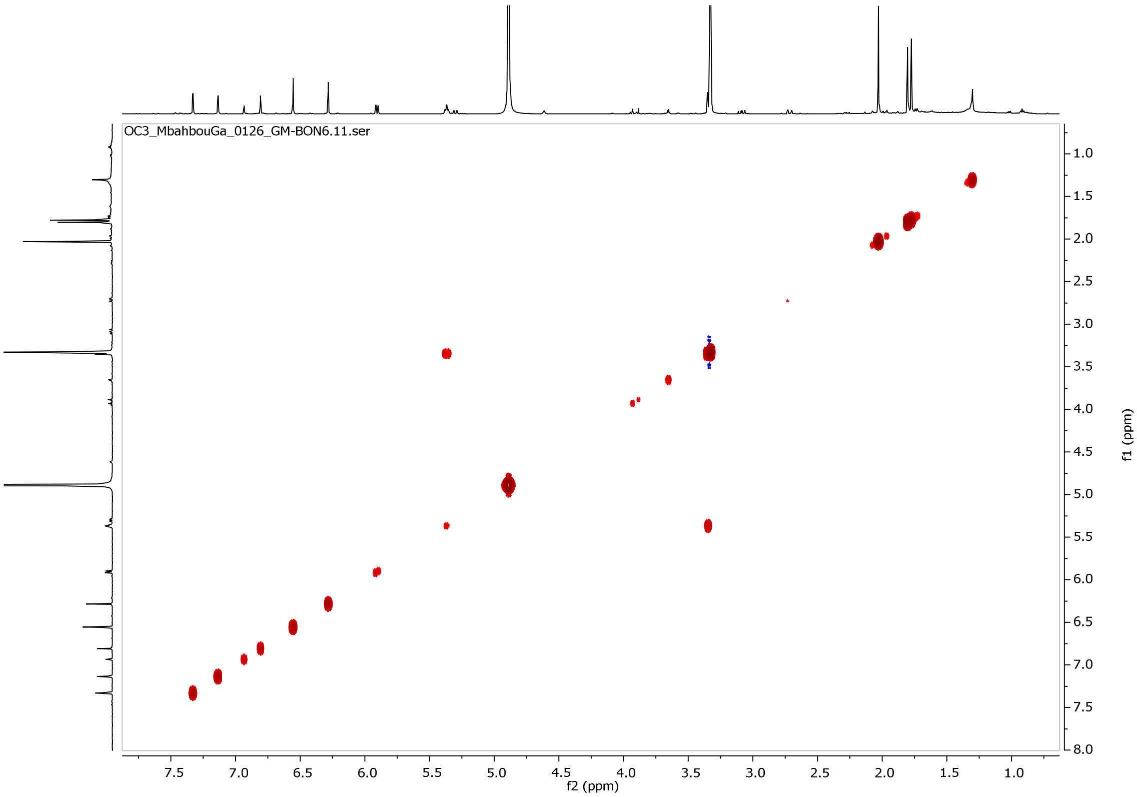

Figure S22. COSY spectrum of compound **3**

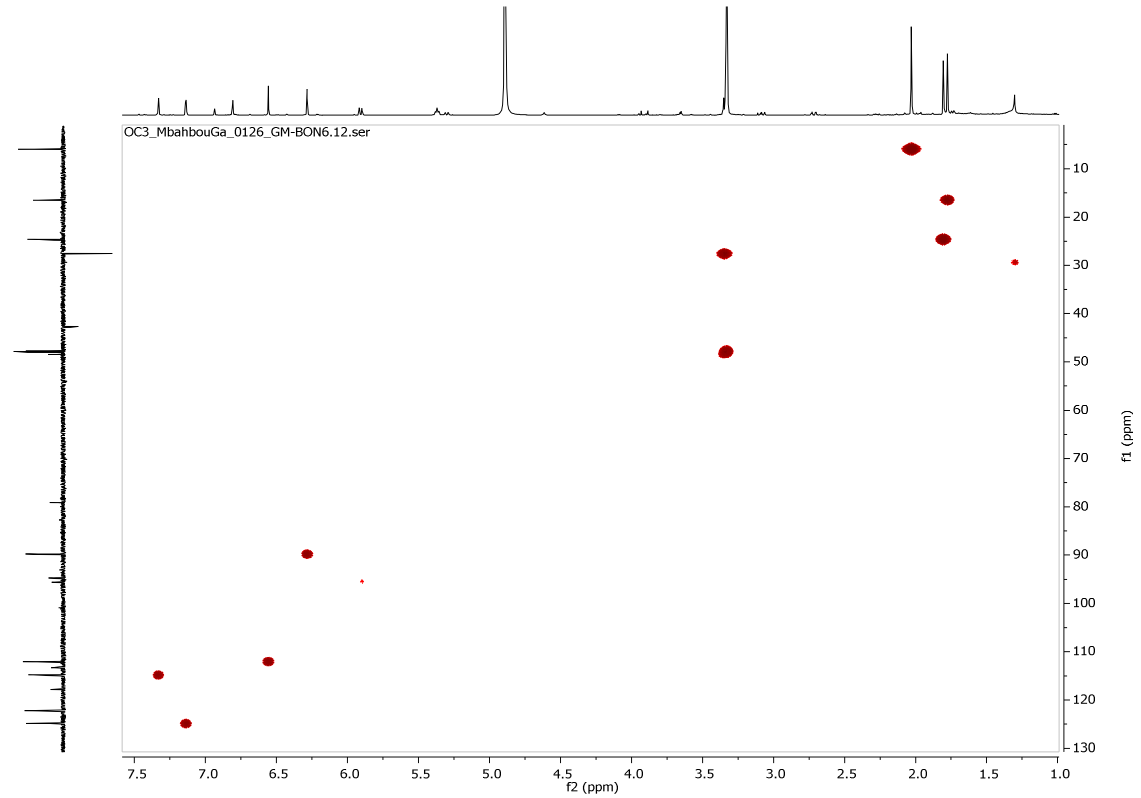

Figure S23. HSQC spectrum of compound **3**

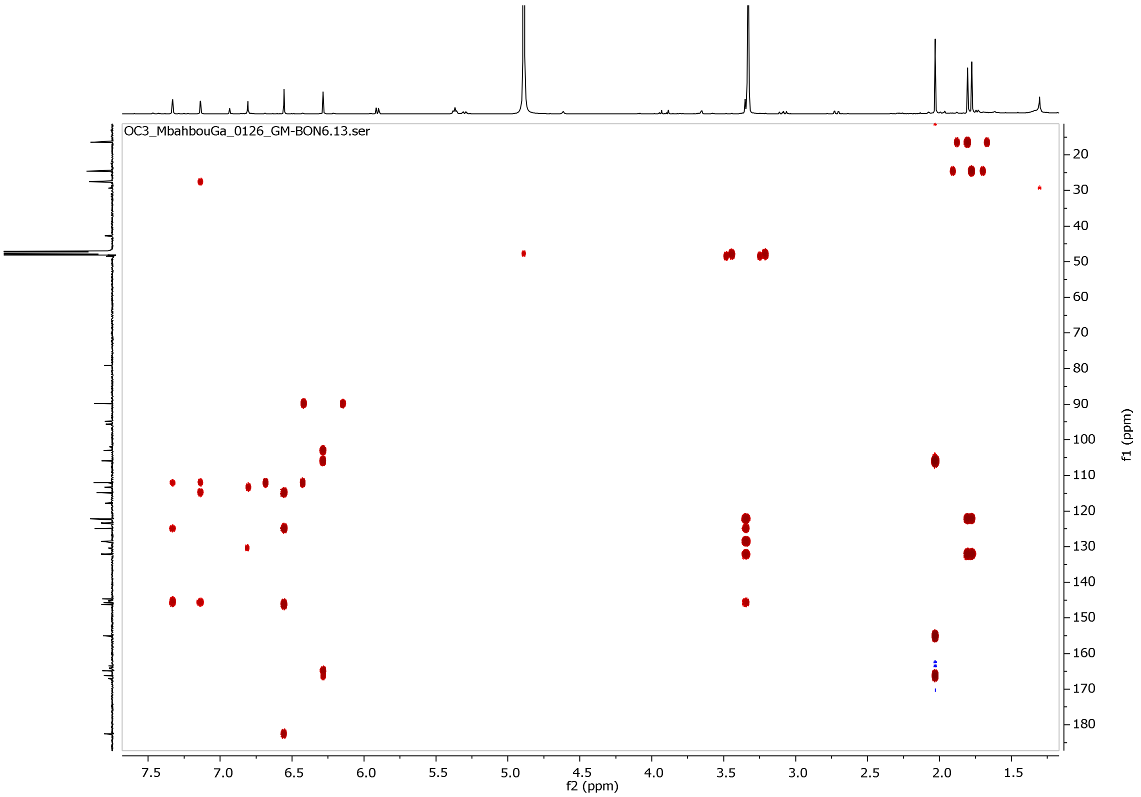

Figure S24. HMBC spectrum of compound **3**

- Mixture of **4** and **5** Nago et al. 2021

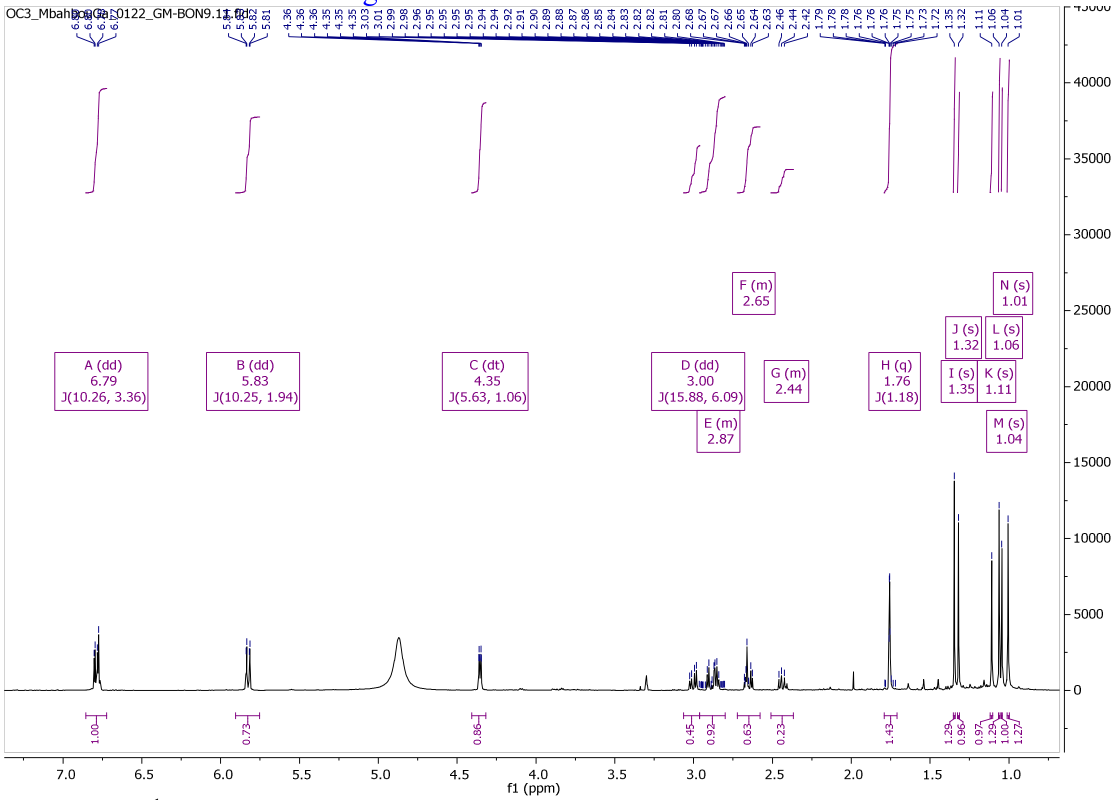

Figure S25. ^{1}H NMR spectrum of mixture of compounds **4+5**

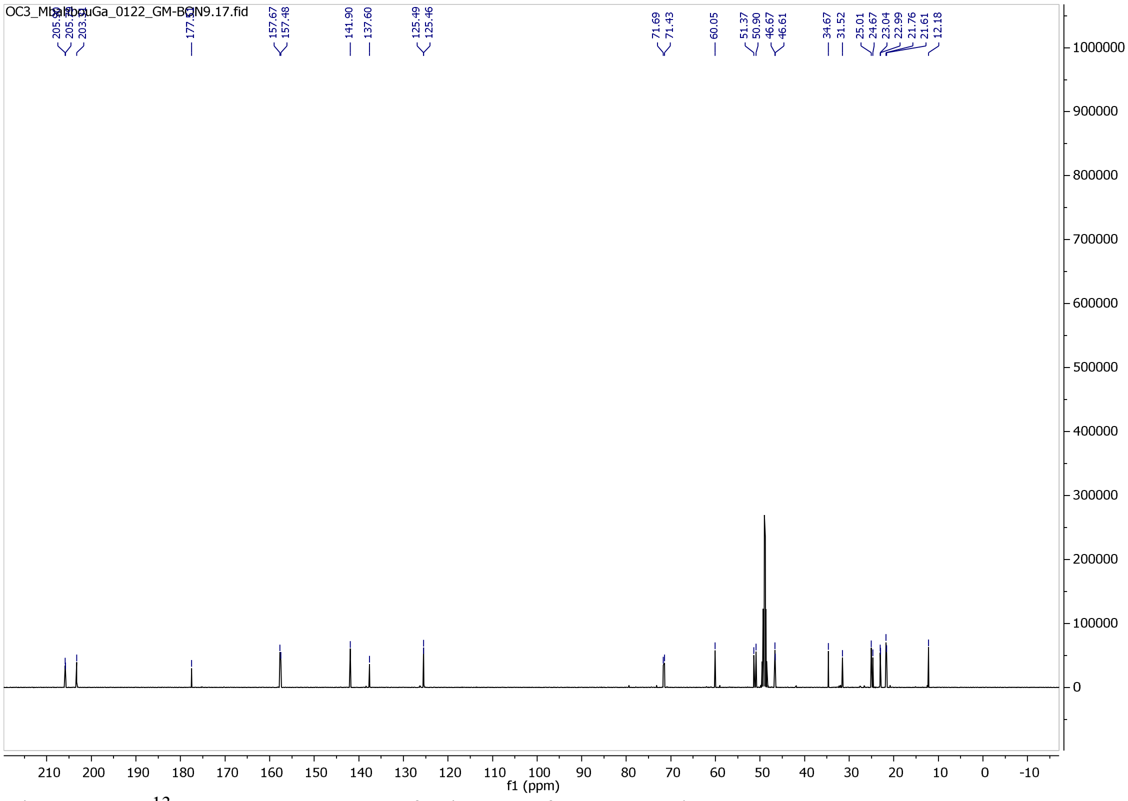

Figure S26. ^{13}C NMR spectrum of mixture of compounds **4+5**

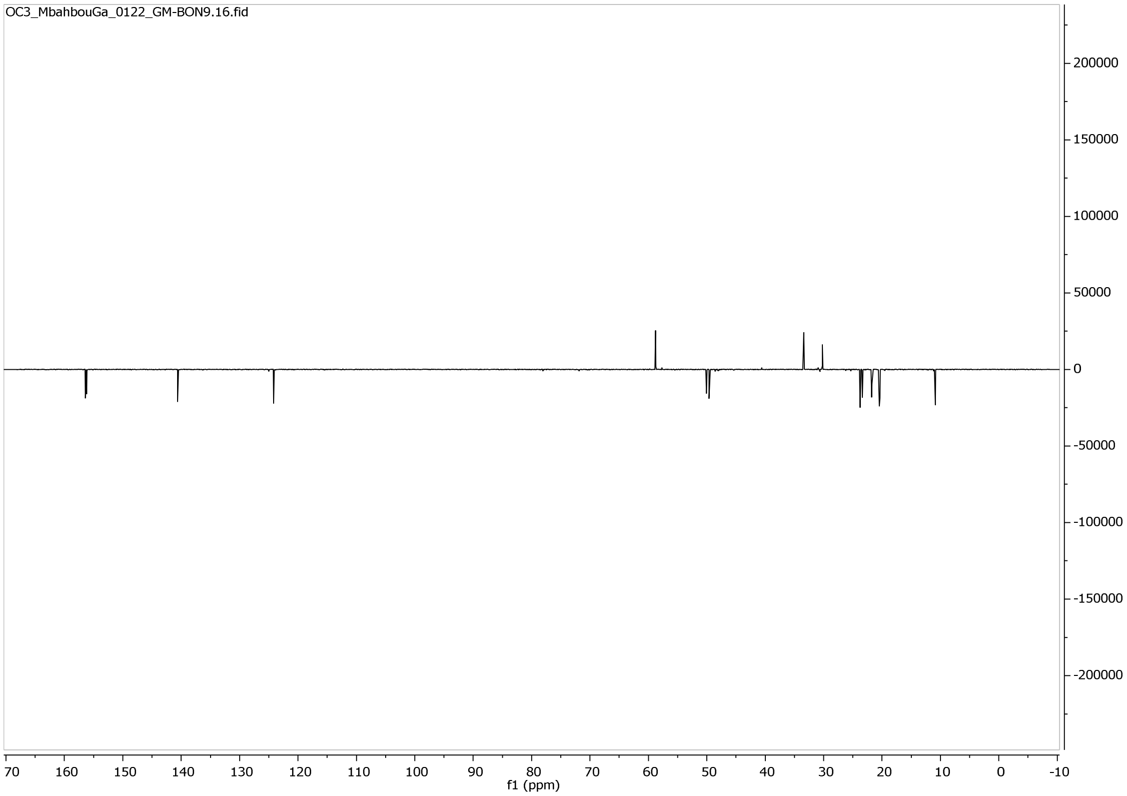

Figure S27. DEPT135 spectrum of mixture of compounds **4+5**

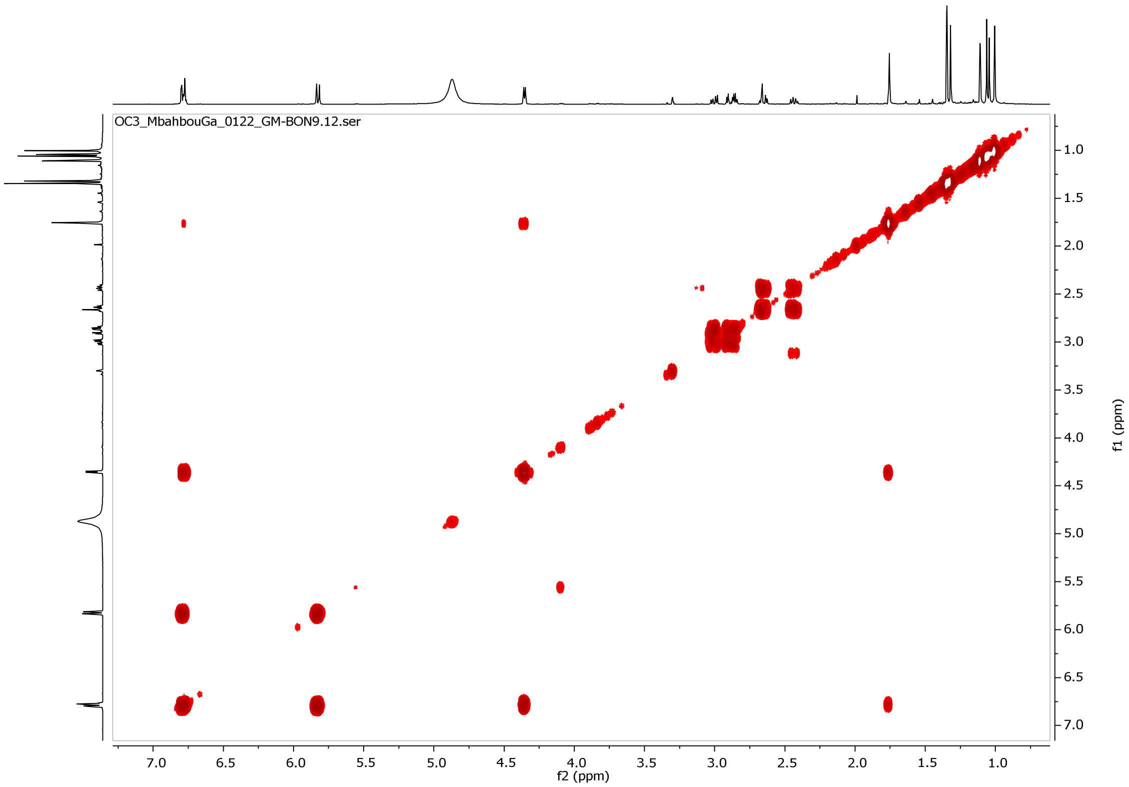

Figure S28. COSY spectrum of mixture of compounds **4+5**

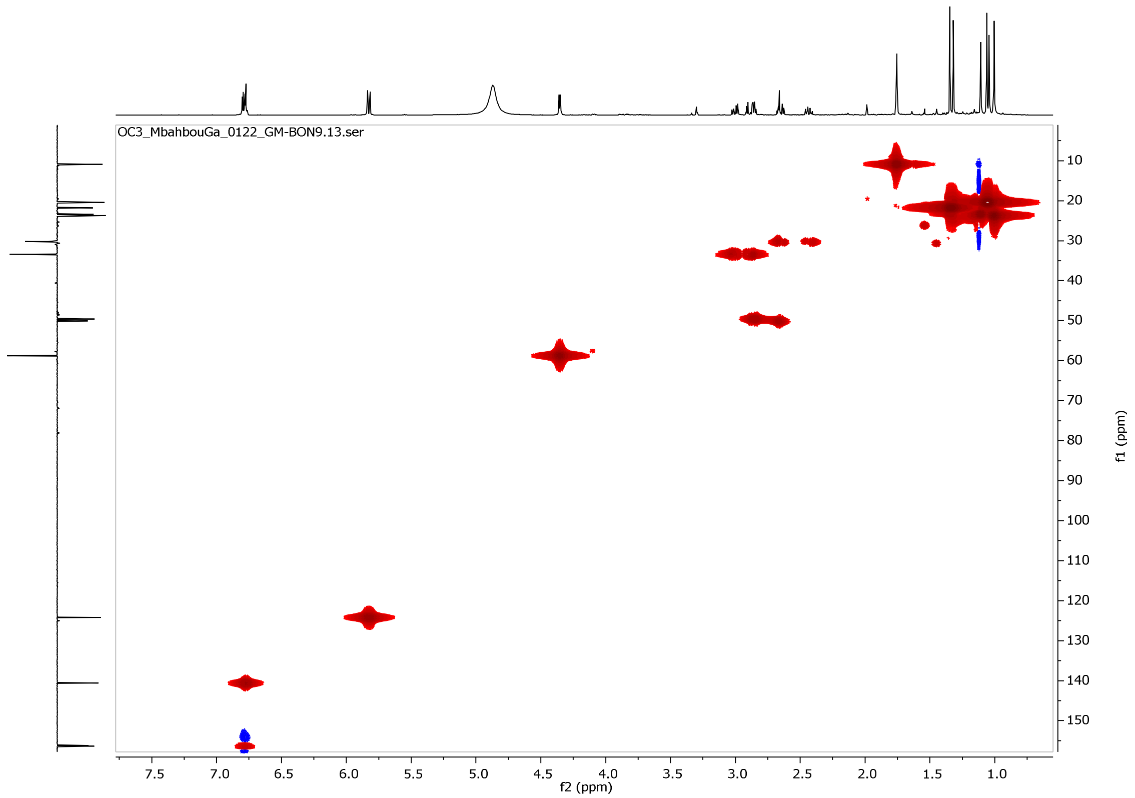

Figure S29. HSQC spectrum of mixture of compounds **4+5**

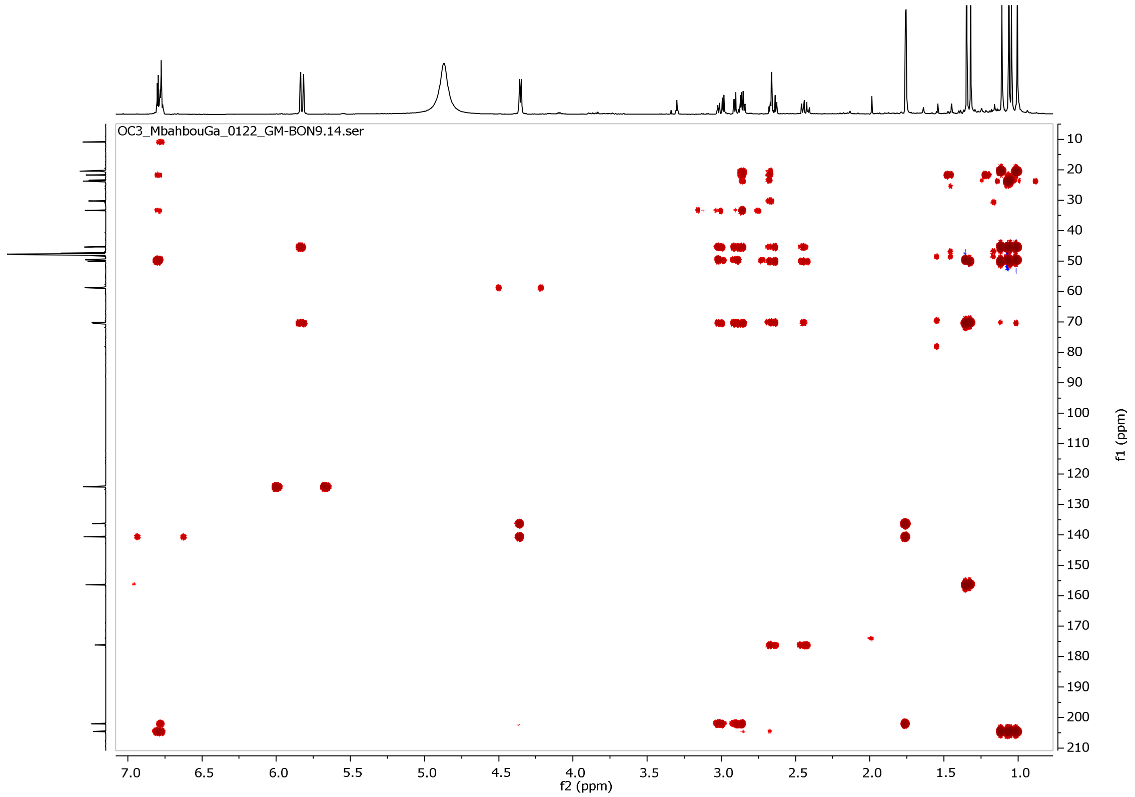

Figure S30. HMBC spectrum of mixture of compounds **4+5**

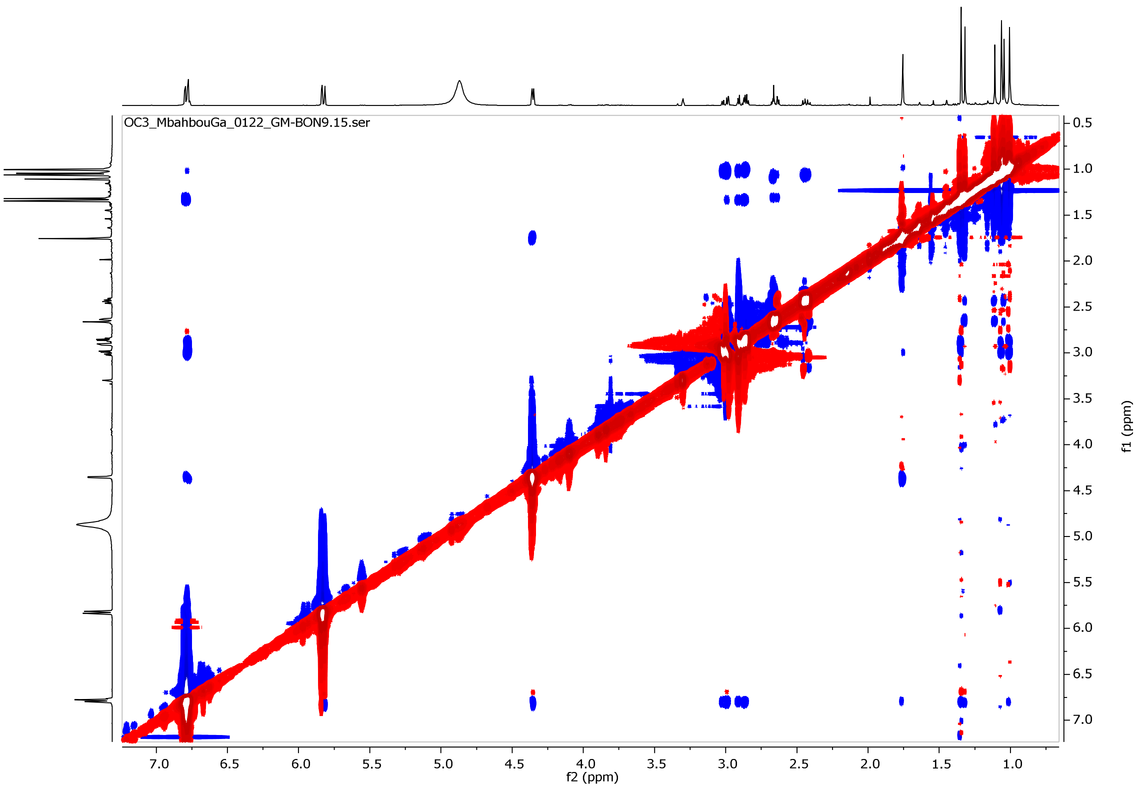

Figure S31. ROESY spectrum of mixture of compounds **4+5**

- Cylindracid A (**4**), Nago et al. 2021

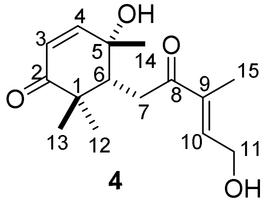

4

Brown oil, *m/z* 266.1, $C_{15}H_{22}O_4$; ^{1}H NMR (MeOD, 500 MHz): δ_H 5.82 (1H, d, J = 10.3 Hz, H-3), 6.78 (1H, d, J = 10.3 Hz, H-4), 2.85 (1H, m, H-6), 2.99 (2H, dd, J = 15.9 and 6.1 Hz, H-7), 6.80 (1H, d, J = 3.4 Hz, H-10), 4.36 (2H, d, J = 5.7 Hz, H-11), 1.11 (3H, s, H-12), 1.05 (3H, s, H-13), 1.34 (3H, s, H-14), 1.75 (3H, s, H-15).
^{13}C NMR (MeOD, 125 MHz): δ_C 46.8 (C-1), 205.8 (C-2), 124.1 (C-3), 156.2 (C-4), 71.9 (C-5), 49.6 (C-6), 33.3 (C-7), 203.4 (C-8), 137.5 (C-9), 140.9 (C-10), 58.9 (C-11), 24.7 (C-12), 21.8 (C-13), 23.0 (C-14), 10.9 (C-15).

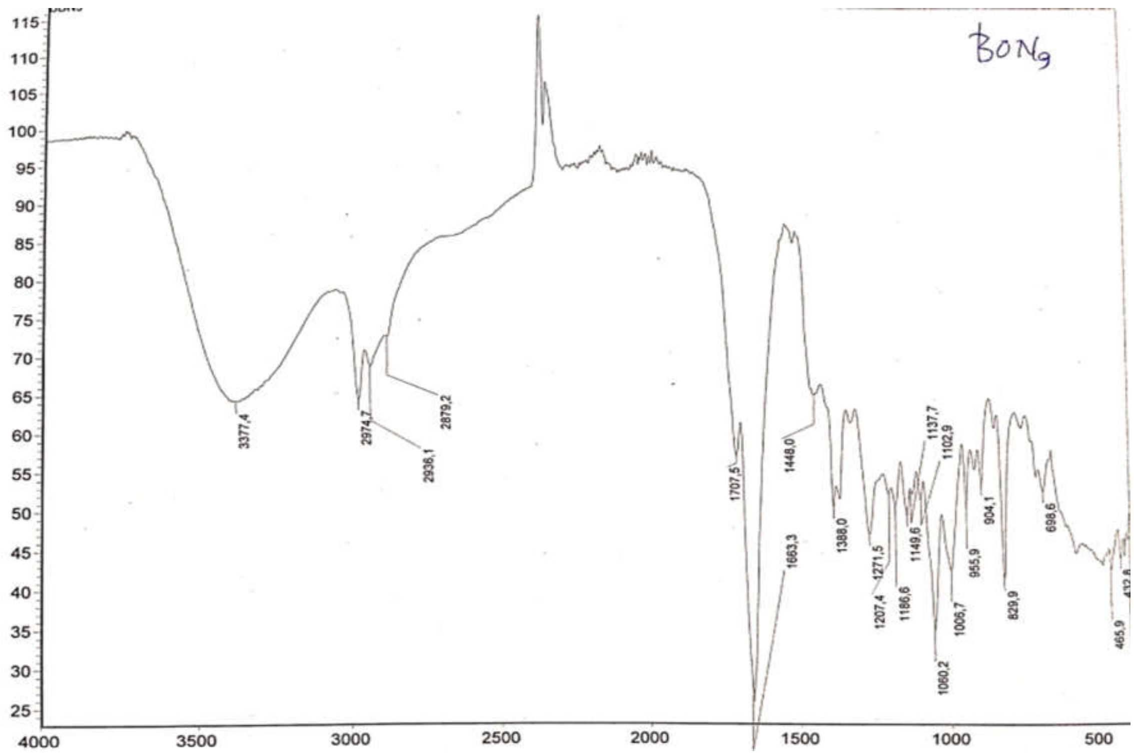

Figure **S32**: IR spectrum of **4**

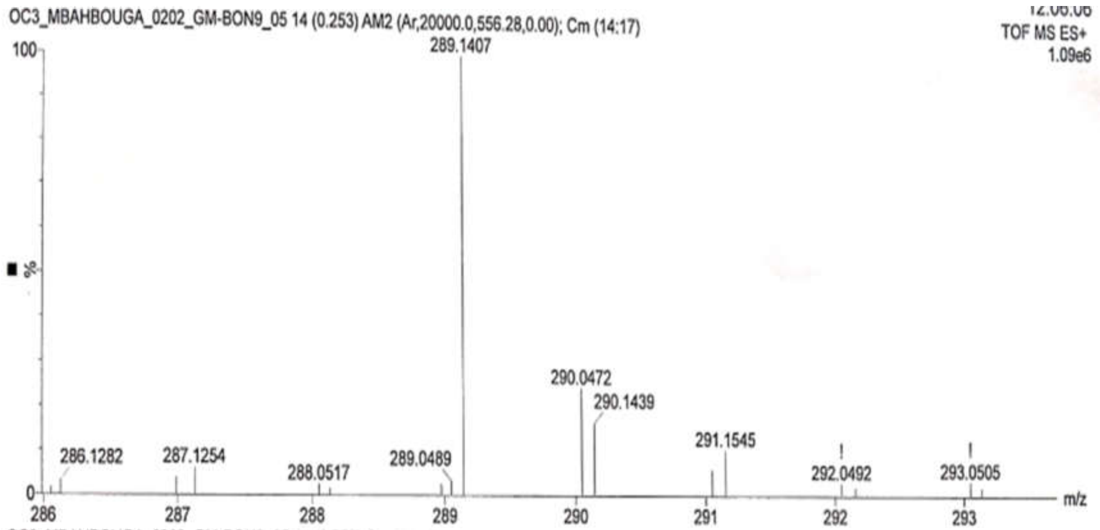

Figure **S33**: **HRESI-MS** spectrum of **4**

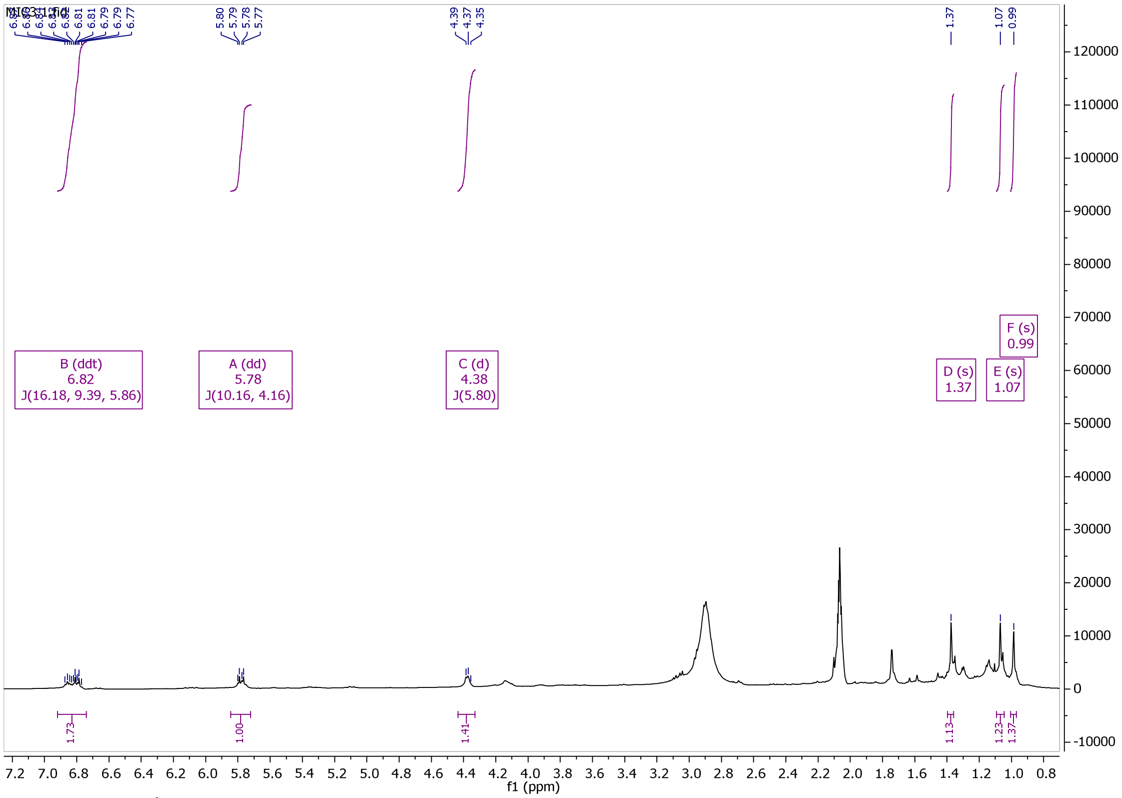

Figure S34. ^{1}H NMR spectrum of compound **4**

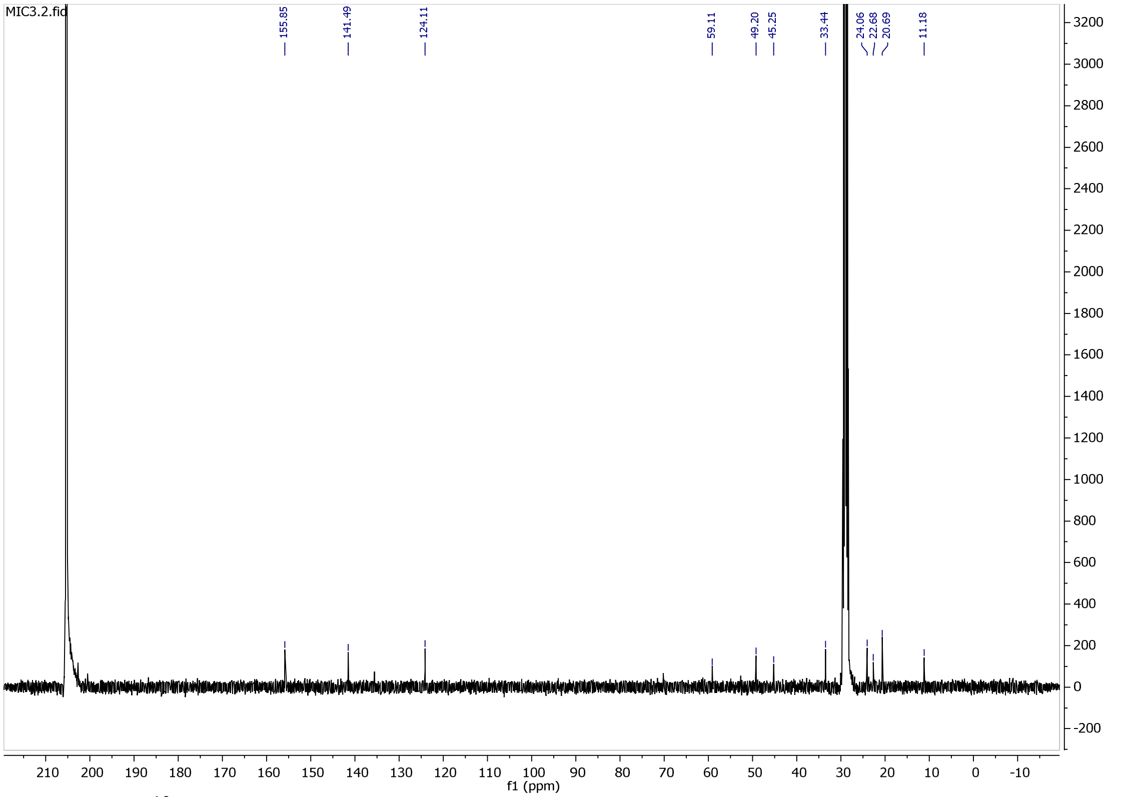

Figure S35. ^{13}C NMR spectrum of compound **4**

- Cylindracid B (**5**), Nago et al. 2021

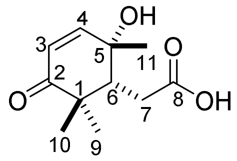

Brown oil, *m/z* 212.1, $C_{11}H_{16}O_4$; ^{1}H NMR (MeOD, 500 MHz): δ_H 5.82 (1H, d J = 10.3 Hz, H-3), 6.78 (1H, d J = 10.3 Hz, H-4), 2.85 (1H, m, H-6), 2.99 (2H, d J = 15.9 and 6.1 Hz, H-7), 1.11 (1H, s, H-9), 1.05 (1H, s, H-10), 1.34 (1H, s, H-11)
^{13}C NMR (MeOD, 125 MHz): δ_C 46.6 (C-1), 205.7 (C-2), 125.4 (C-3), 157.4 (C-4), 71.4 (C-5), 51.3 (C-6), 34.6 (C-7), 177.5 (C-8), 25.0 (C-9), 21.7 (C-10), 23.1 (C-11)

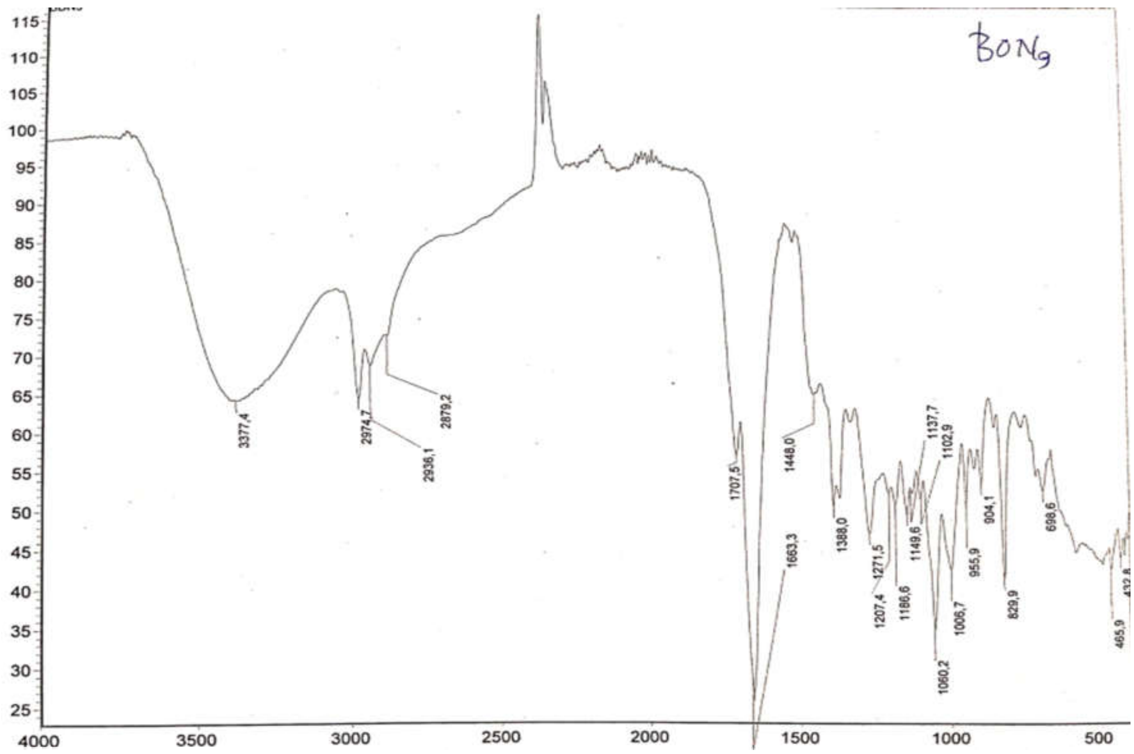

Figure **S36**: IR spectrum of **5**

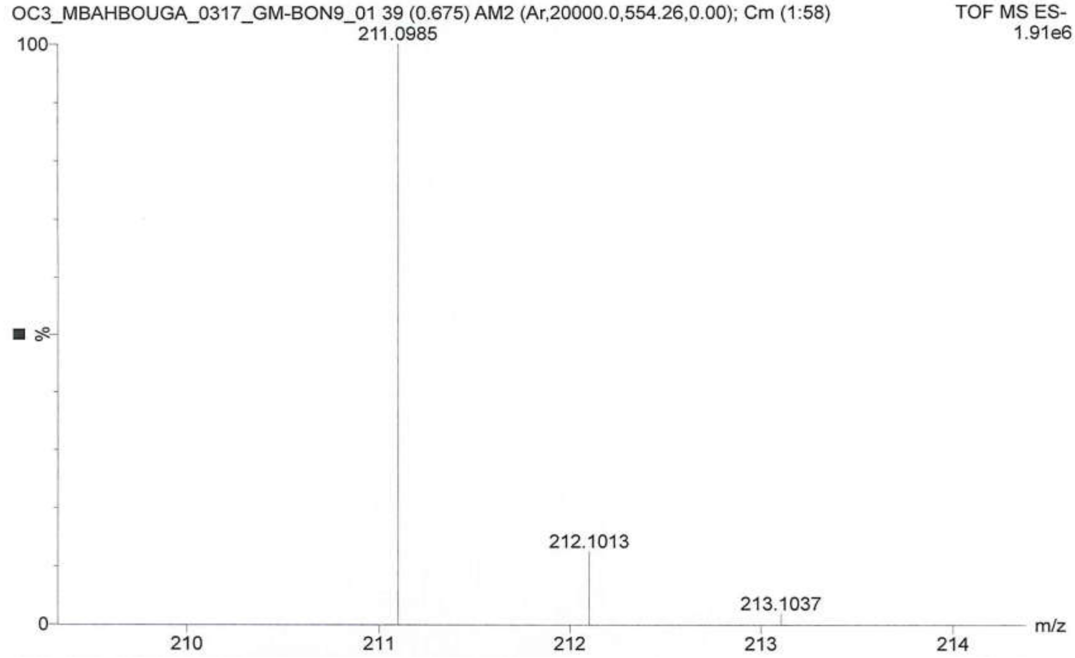

Figure **S37**: **HRESI-MS** spectrum of **5**

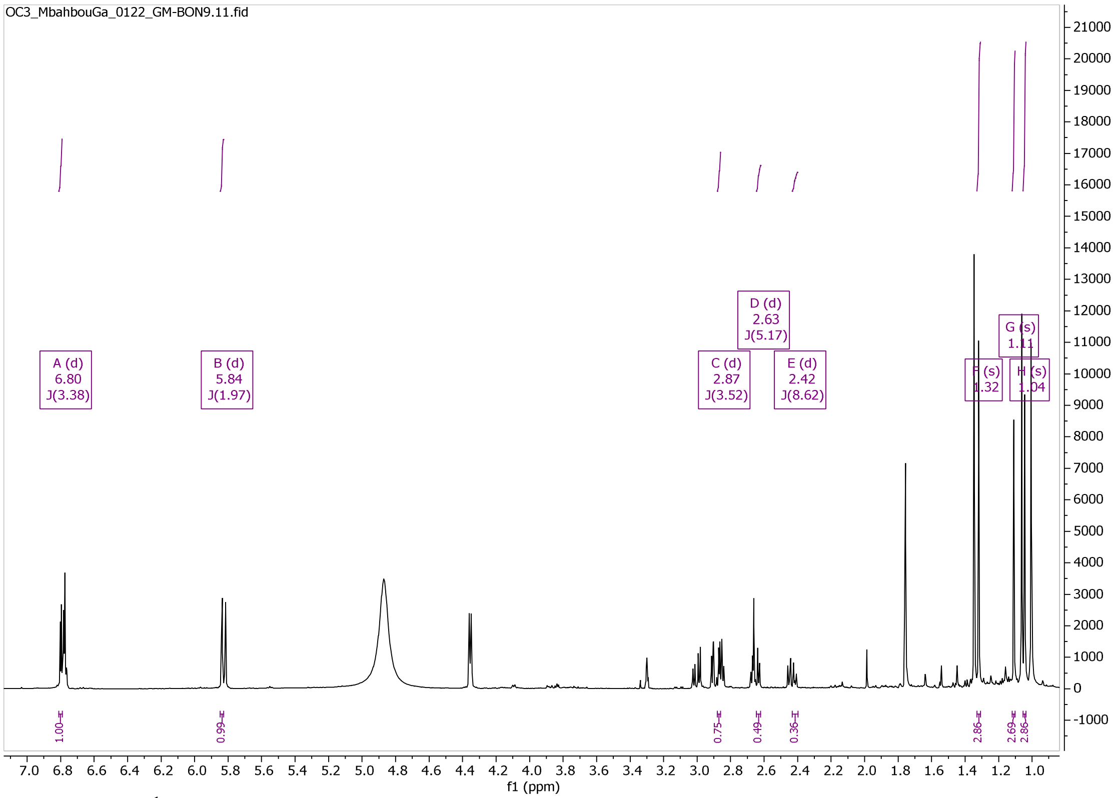

Figure S38. ^{1}H NMR spectrum of mixture of compounds **4**+**5** with indicated signals of compound **5**

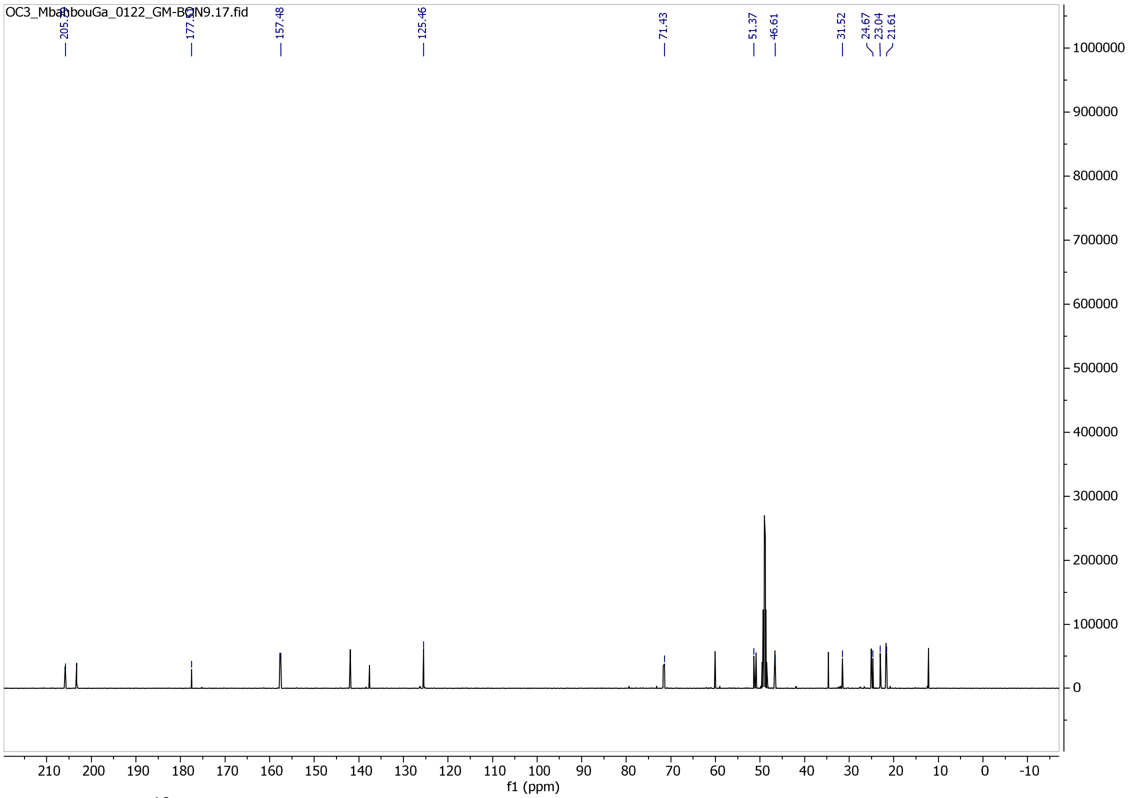

Figure S39. ^{13}C NMR spectrum of mixture of compounds **4+5** with indicated signals of compound **5**
